# Nano‐Anesthetics Regulate Neuro‐Immune Interaction for Treating Neuropathic Pain

**DOI:** 10.1002/advs.202502920

**Published:** 2025-05-21

**Authors:** Yue Wang, Xiuru Ji, Yu Sun, Han Wang, Ting Wang, Tao Luo, Yanyong Cheng, Jia Yan, Dalong Ni, Hong Jiang

**Affiliations:** ^1^ Department of Anesthesiology Shanghai Ninth People's Hospital Shanghai Jiao Tong University School of Medicine Shanghai 200011 P. R. China; ^2^ Department of Orthopaedics Shanghai Key Laboratory for Prevention and Treatment of Bone and Joint Diseases Shanghai Institute of Traumatology and Orthopaedics Ruijin Hospital Shanghai Jiao Tong University School of Medicine Shanghai 200025 P. R. China; ^3^ Department of Biomaterials and Stem Cells Suzhou Institute of Biomedical Engineering and Technology Chinese Academy of Science Suzhou 215163 P. R. China

**Keywords:** metal–organic framework, nanomedicine, neuro‐immune interaction, neuropathic pain

## Abstract

Neuropathic pain is a multifaceted syndrome posing significant challenges to patient quality of life and healthcare systems. Conventional treatments primarily focus on general pain modulation, which fail to address specific underlying mechanisms, leading to limited efficacy and infinite side effects. Calcitonin gene‐related peptide (CGRP) has played a pivotal role in neuro‐immune repair, contributing to vasodilation, nociception, and immune modulation following tissue injury. Herein, a bupivacaine‐loaded cerium‐based metal–organic framework (CUB) is designed to integrate sustained release of analgesia with immunomodulatory and antioxidant capabilities. In vivo models of chronic constriction injury (CCI) have demonstrated that CUB significantly reduced neuroinflammation, promoted M2 microglial polarization, and enhanced myelin regeneration for the prolonged analgesia. Deep mechanism analysis revealed that the designed CUB can significantly elevate TSP‐1 expression to activate CGRP signal in modulating the neuro‐immune interaction, contributing to the repair process. Notably, the CUB outperformed standalone bupivacaine or cerium nanoparticles in terms of pain relief, motor function recovery, and neuroglial regulation. The findings highlight the potential of CUB as a multifactorial therapeutic for treating neuropathic pain, offering new perspectives on the integration of nanotechnology in chronic pain management through neuro‐immune pathways.

## Graphical abstract

Ce‐UiO‐66‐Bupivacaine (CUB) demonstrates triple therapeutic innovation: 1) Sustained analgesia with ROS scavenging synergistically resolves neuropathic pain; 2) Microglia/astrocyte polarization switch reprograms the pro‐regenerative immune microenvironment; 3) CGRP‐TSP‐1 axis activation couples inflammation suppression to axon regeneration, defining a new neuromodulation paradigm.

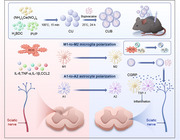

## 1 Introduction

Neuropathic pain refers to a range of complex and multifaceted syndromes, imposing significant burdens on society, healthcare systems, and personal well‐being.^[^ ^1^, ^2^ ^]^ Statistics estimate that the global incidence of neuropathic pain ranges between 7% and 10%, with direct medical expenses in the United States amounting to $10 billion annually and 14 billion yuan in China, both showing an upward trend.^[^ ^3^ ^]^ Standard first‐line treatment options include five categories of oral medication (i.e., serotonin‐/norepinephrine‐modulating antidepressants, sodium‐channel blocking anticonvulsants, calcium‐modulating anticonvulsants, tramadol, and opioids) and two classes of topical medications (i.e., local anesthetics and capsaicin). However, these drugs fail to specifically target the underlying mechanisms of neuropathic pain, acting instead through general pain modulation and neuronal inhibition, which often results in systemic side effects, such as respiratory depression, cardiotoxicity, immunosuppression, blood pressure dysregulation, and cognitive impairment. Thus, pursuing effective and targeted therapies or interventions that can fundamentally alleviate pain and restore motor function is of both scientific and social importance.^[^ ^4^, ^5^ ^]^

Although the precise aetiological causes of neuropathic pain remain unclear, it has been established that neuro‐immune imbalance plays important roles in the occurrence and progression of neuropathic pain. Under normal conditions, the nervous system plays a critical role in maintaining immune homeostasis. However, during nerve injury, the immune system becomes instrumental by releasing molecular mediators that sensitize nociceptor neurons. Tissue injury and inflammation are closely linked to an enhanced pain response. Nociceptor terminals in the periphery, which contain specific receptors and ion channels, are responsible for detecting these mediators released during inflammation. Under pathological conditions, the activation of these receptors initiates action potentials, which are then transmitted to nociceptor cell bodies located in the dorsal root ganglia (DRG). These signals are then relayed to the spinal cord and brain, where they are processed and perceived as pain.^[^ ^6^, ^7^ ^]^ Moreover, excessive reactive oxygen species (ROS) and neuroinflammation exacerbate pain and nerve damage,^[^ ^8^ ^]^ and the difficulty of restoring homeostasis to an already compromised neuroimmune microenvironment amplifies the inflammatory response, creating a vicious cycle.

Bidirectional interactions between neurons and glial cells, including microglia and astrocytes, as well as reciprocal interactions between neurons and immune cells such as T cells and macrophages, have been identified as key contributors to neuropathic pain pathogenesis.^[^ ^9^, ^10^, ^11^ ^]^ The significant polarization of microglial cells observed in the dorsal horn of the spinal cord following peripheral nerve injury may be attributed to two main mechanisms: the infiltration of circulating monocytes and the self‐renewal of resident microglia and astrocytes.^[^ ^12^, ^13^ ^]^ It is hypothesized that activated glial cells exert a pivotal influence on neuronal excitability and synaptic plasticity in response to neurological disorders. Furthermore, they are postulated to play a pivotal role in the transformation of peripheral nerve injury into central spinal sensitization and neuropathic pain. The dialogue between nociceptors and the immune system is therefore a fundamental aspect in both acute and chronic inflammation.^[^ ^14^, ^15^, ^16^ ^]^ Approaches that regulate these interactions are expected to benefit the treatment of neuropathic pain.

**Figure 1:**
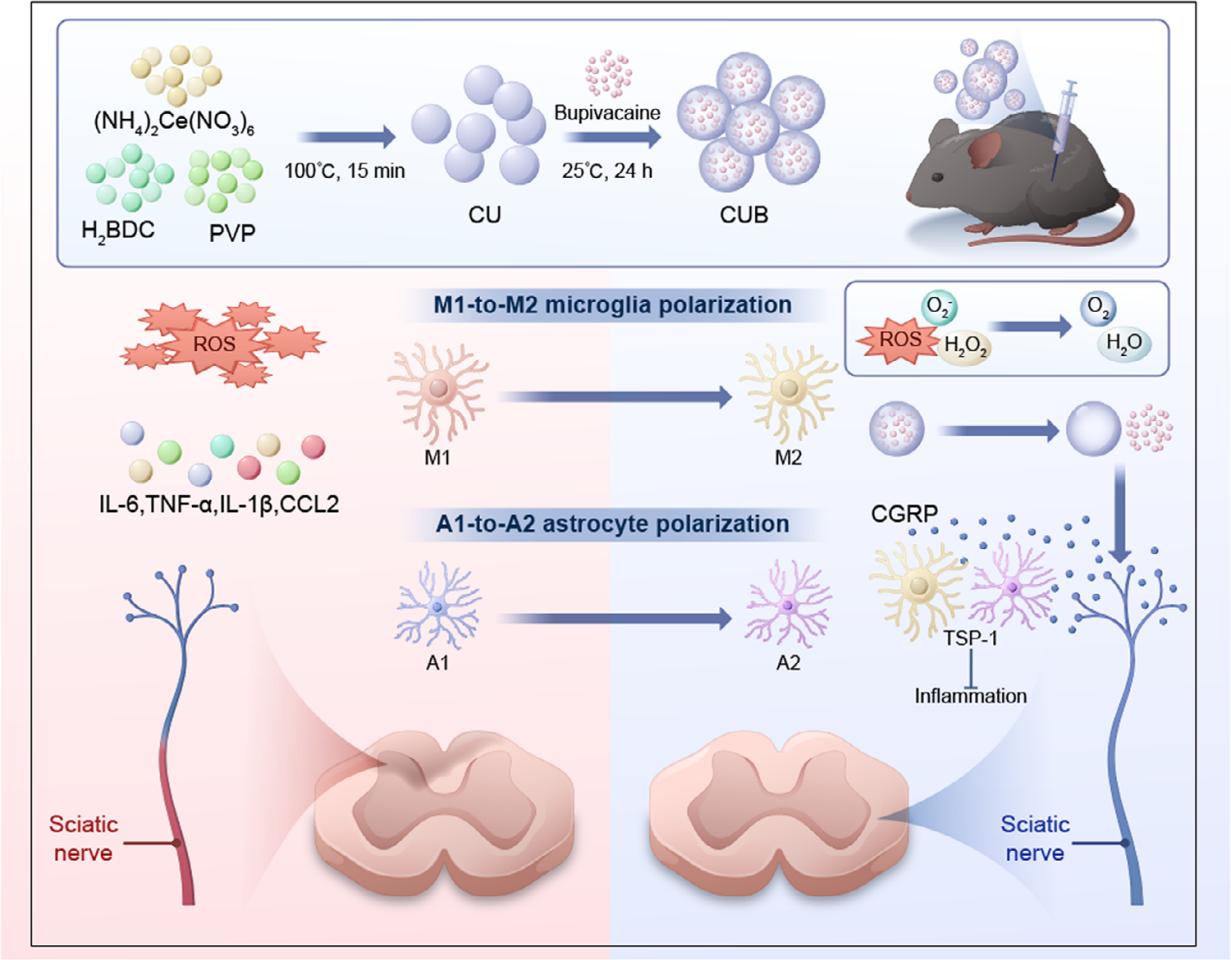
Schematic diagram of the application and proposed mechanisms of CUB. Following the treatment with CUB on the CCI mice model, the nerve environment is regulated to a favorable state of immune tolerance through the administration of effective long‐term analgesia, alleviation of neuroinflammation, and removal of excess reactive oxygen species. In addition, CUB promotes nerve repair and inhibits neuroinflammation via controlling microglia and astrocyte polarization. CGRP signaling induces the release of the multifunctional extracellular matrix protein TSP‐1, leading to the co‐suppression of inflammation and promotion of nerve regeneration.

Metal–organic frameworks (MOFs) have recently emerged as promising nanomaterials for drug delivery, characterized by their chemical tunability, high surface area, and pore size, allowing for efficient loading of therapeutic cargo and sustained release.^[^ ^17^, ^18^, ^19^, ^20^, ^21^, ^22^ ^]^ Herein, a neuro‐immune interaction regulation strategy was first proposed for neuropathic pain treatment, achieved by combining bupivacaine and Ce‐UiO‐66 (abbreviated as CUB). The Ce‐UiO‐66 acts as an antioxidant by scavenging ROS overproduced during nerve injury, while the sustained‐release bupivacaine provides prolonged analgesic effects for up to 24 h—significantly longer than the 6‐h action of bupivacaine alone. Furthermore, the CUB induces microglia in the spinal cord to generate M2‐like (anti‐inflammatory) phenotypes, thereby acting as adaptive immune modulators (**Figure** **1**). Overall, as an enhanced sustained release system with distinguished immune modulation and ROS scavenging properties, the designed CUBs demonstrate the effectiveness of a multifactor‐based strategy for treating neuropathic pain, which will open up new perspectives for the development of nanotechnology in chronic pain management.

## 2 Results and Discussion

### 2.1 Synthesis and Characterization of Ce‐UiO‐66‐Bupivacaine (CUB)

**Figure 2:**
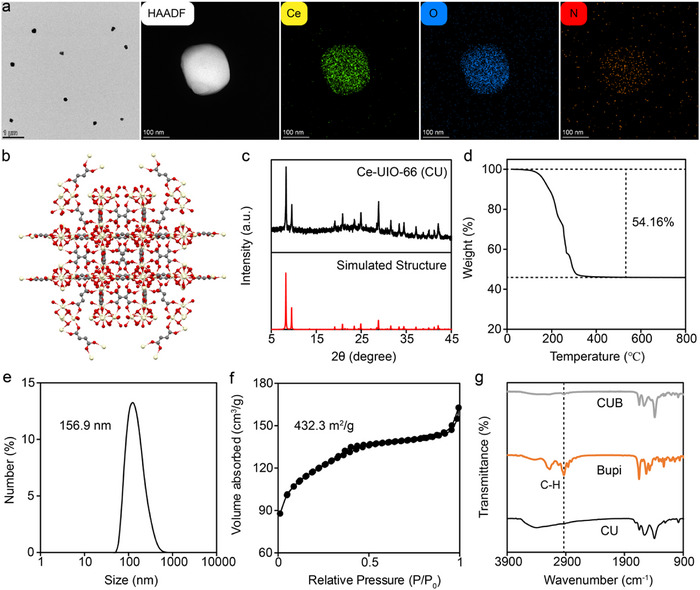
Characterization of materials. a) TEM, HAADF‐STEM, and elemental mapping images of CU. b) Illustration of CU structure. c) XRD pattern of CU. d) Thermogravimetric analysis of CU. e) Hydrodynamic diameters of CU. f). Nitrogen adsorption‐desorption isotherms of CU. g) FTIR spectra of CU, Bupi, and CUB.

The Ce‐UiO‐66 (CU) was synthesized according to previous research.^[^ ^23^ ^]^ Subsequently, a solution of bupivacaine (Bupi) was added to the CU solution to obtain Ce‐UiO‐66‐Bupivacaine (CUB). Transmission electron microscopy (TEM) and high‐angle annular dark field scanning transmission electron microscopy (HAADF‐STEM) image (**Figure** **2**a) presented the nanoparticle morphology of CU. The elemental mapping (Figure 2a) showed the co‐existence of Ce, O, and N atoms in CU. The structure of CU was shown in Figure 2b, which was in accordance with the simulated structure, as proved by X‐ray diffraction (Figure 2c). Thermogravimetric analysis (TGA) showed a mass loss of 54.16% of CU (Figure 2d), and the thermal decomposition product of CU was ceria, which theoretically would cause 38.1% mass loss. Hence, the extra mass loss could be attributed to PVP. Dynamic light scattering (DLS) measurements showed the hydrodynamic diameters of CU were 156.9 nm, and the size distribution remained consistent over time, with no significant aggregation or degradation observed over 14 days. (Figure 2e; Figure S1, Supporting Information). The surface area of CU was measured to be 432.3 m^2^ g^−1^ (Figure 2f), which could be used for drug delivery. Fourier transform infrared (FT‐IR) spectroscopy (Figure 2g) showed that both Bupi and CUB contained the carbon‐hydrogen bond absorption peak (2930 cm^−1^), along with the UV–vis data showed a characteristic peak at 245 nm corresponding to bupivacaine (Figure S2a, Supporting Information), indicating that CUB was successfully loaded with Bupi. Based on these measurements, we calculated a drug loading efficiency of ≈85% and an encapsulation efficiency of ≈90%. In vitro release studies further revealed that under simulated tissue injury conditions (pH 6.0), ≈90% of bupivacaine was released within 8 h, while only ≈26% was released at physiological conditions (pH 7.4) over the same period (Figure S2b, Supporting Information). These findings not only demonstrate the efficient loading of bupivacaine into CU but also highlight the pH‐responsive release behavior, which is particularly relevant for targeting injured tissue. The zeta potential results demonstrated that bupivacaine loading converts the surface charge of CU from –9.93 mV to +28.2 mV, confirming successful drug incorporation and potentially enhancing cellular uptake and in vivo efficacy (Figure S3, Supporting Information). The above results demonstrated the successful synthesis of CUB.

### 2.2 ROS‐Scavenging Activity and Anti‐Inflammatory Effect of CUB

Inflammatory responses can trigger ROS production as part of the immune system's defense mechanism. The generated ROS will act as signaling molecules that promote inflammation and amplify immune responses. We first conducted hydrogen peroxide decomposition assays and total antioxidant capacity assays to evaluate the overall antioxidant activity of CUB. Both CU and CUB exhibited remarkable antioxidant ability. When the concentration of CU and CUB was up to 40 ppm, nearly 76% oxidation reaction could be inhibited (Figure S4a, Supporting Information). Then, the ability of CU and CUB to scavenge ROS, such as H_2_O_2,_ was evaluated. At 20 ppm, CU and CUB were found to decompose ≈85% of the total H_2_O_2_ present in the solution and minimal tissue toxicity, indicating that the intrinsic Ce^3^⁺/Ce⁴⁺ redox cycling responsible for ROS scavenging is retained upon bupivacaine incorporation (Figure S4b,c, Supporting Information). This concentration also ensured that the bupivacaine loading was sufficient to achieve the desired analgesic effect (Figure S4d, Supporting Information). These results indicated that the CUB had effective antioxidant capacity, and the addition of bupivacaine did not influence the antioxidant ability of CU.

**Figure 3:**
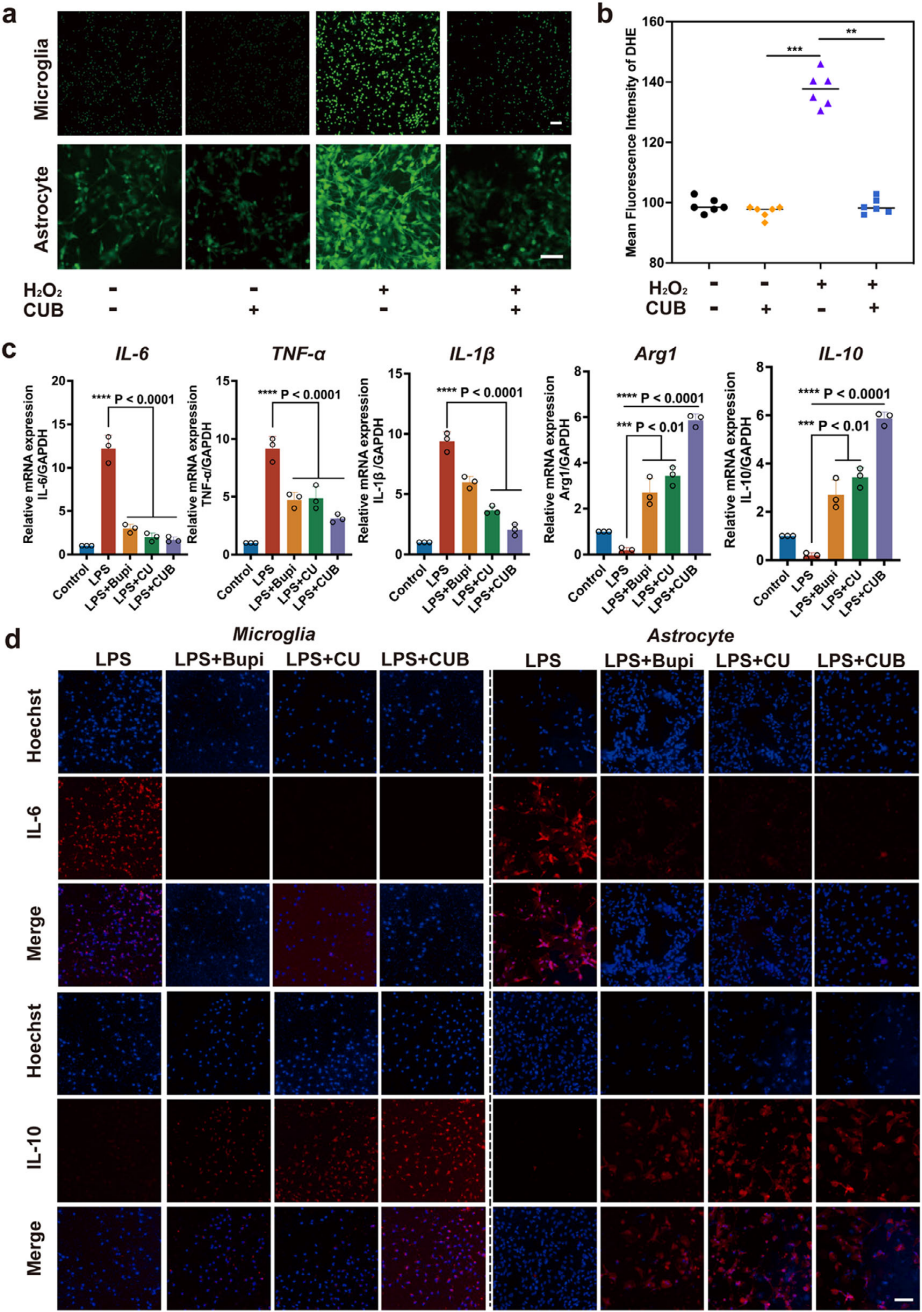
ROS‐scavenging activity and protective effects of CUB in neuroglia. a,b) In vitro ROS‐scavenging of CUB confirmed by confocal microscopy and the corresponding quantifications (n = 6). c) Relative mRNA expression levels of inflammatory molecules (IL‐6, TNF‐α, IL‐1β) and anti‐inflammatory molecules (IL‐10 and Arg1) in microglia treated with different groups (i.e., control, LPS, LPS+Bupi, LPS+CU, LPS+CUB) measured by quantitative PCR with reverse transcription (qRT–PCR). Data are mean ± s.e.m. (n = 3). d) Representative staining for inflammatory factor (IL‐6) and anti‐inflammatory factor (IL‐10) in different groups. *P < 0.05, **P < 0.01, ***P < 0.001, ****P < 0.0001. All scale bars = 100 µm.

Microglia and astrocytes were treated with H_2_O_2_ or lipopolysaccharide (LPS) to induce an oxidative stress model and a neuroinflammation model, respectively. The toxic effects of CU and CUB on microglia and astrocytes were examined prior to subsequent experiments. The results demonstrated that both CU and CUB exhibited minimal cytotoxicity and did not affect cell viability (Figures S5–S7, Supporting Information). Given that excessive ROS production is a primary driver of neuroinflammation and glial activation, we assessed the in vitro ROS‐scavenging capacity of CUBs. Confocal microscopy images showed that CUBs effectively scavenged the overproduced ROS in both microglia and astrocytes (**Figure** **3**a,b).

Based on these findings, the inflammatory cytokine expression under neuroinflammatory conditions was measured. The mRNA expression of inflammatory molecules (IL‐6, TNF‐α, IL‐1β) was significantly decreased, while the mRNA expression of anti‐inflammatory molecules (IL‐10 and Arg1) was significantly increased in the Bupi, CU, and CUB treatment groups when compared with the LPS group (Figure 3c,d). Although both the Bupi and CU treatment groups individually demonstrated anti‐inflammatory effects, their efficacy was less pronounced compared to CUB. This suggests that the catalytic properties of Ce and the anti‐inflammatory effects of bupivacaine act synergistically to modulate the innate immune response. Taken together, these findings indicate that CUBs hold promise as effective ROS scavengers in the treatment of peripheral neuropathic pain.

### 2.3 In Vivo Analgesic Effects and Neuromotor Functional Recovery of CUB

**Figure 4:**
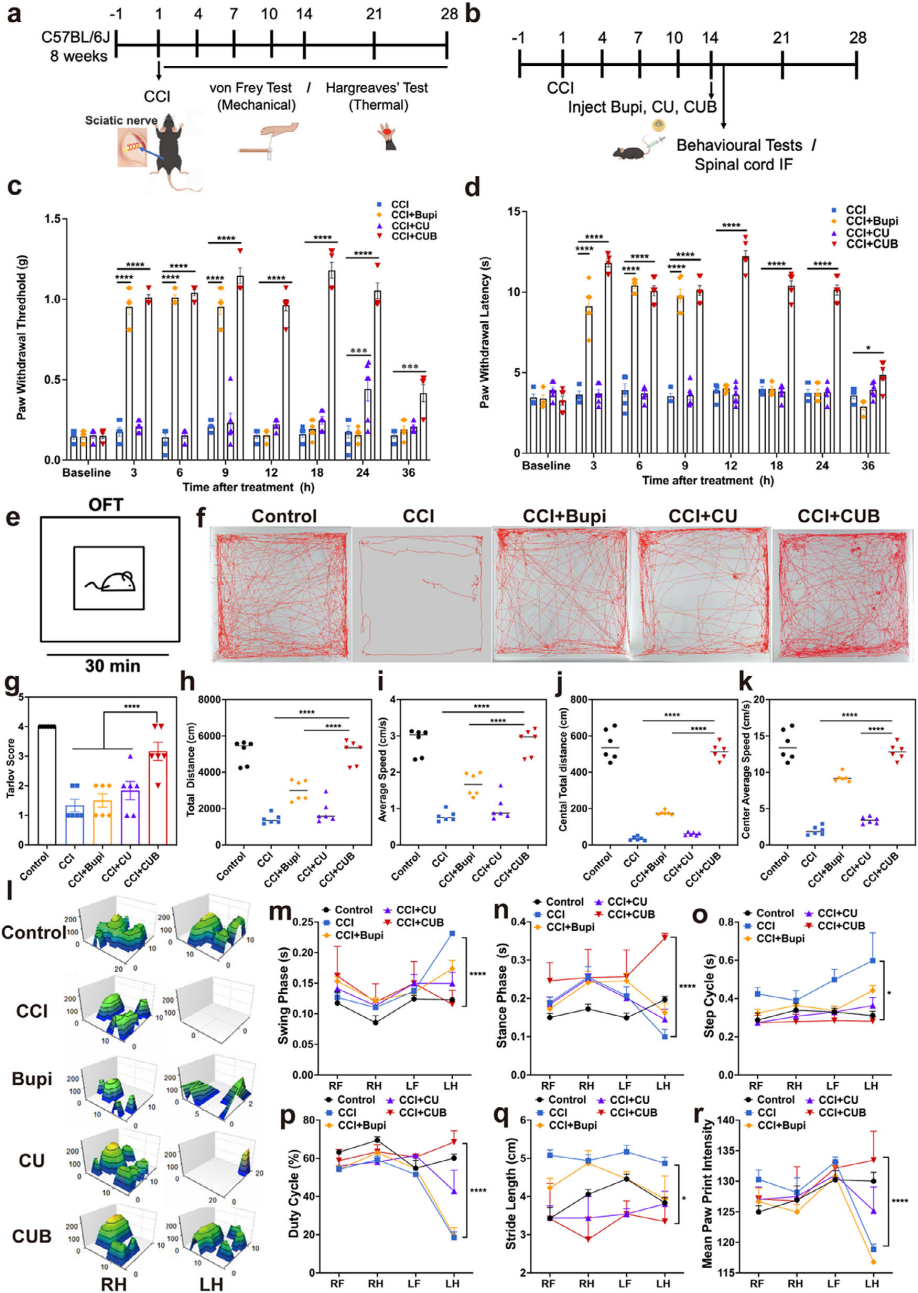
Analgesic effects and neuromotor functional reconstruction at the 3^rd^ week after surgery. a,b) Schematic diagram of modeling, behavioral testing, and treatment. Five groups, including Control, CCI, CCI+Bupi, CCI +CU, and CCI+CUB. c) Mechanical paw withdrawal threshold before and after different treatments. d) Thermal paw withdrawal latency before and after different treatments. Data are mean ± s.e.m.; n = 6 per group. e) Scheme illustration of OFT experiments schedule. f) Representative trace images from the OFT in Control, CCI, CCI+Bupi, CCI+CU, and CCI+CUB groups, respectively. g) Tarlov Score of five groups. h–k) Total and central distances traveled (cm) and average speed (cm/s) over a 30 min duration in different groups (n = 6 mice per group). l) Gait pressure analysis was performed on the mice's right front (RF), right hind (RH), left front (LF), and left hind (LH) paws across the Control, CCI, CCI+Bupi, CCI+CU, and CCI+CUB groups. m–r) Measurements of the swing phase, stance phase, step cycle, duty cycle, stride length, and mean paw print intensity for the RF, RH, LF, and LH paws were recorded for all five groups. All data are expressed as the mean ± s.e.m. *P < 0.05; **P < 0.01; ***P < 0.001, ****P < 0.0001.

To examine the analgesic effect of CUB on peripheral neuropathic pain, a chronic constriction injury (CCI) model of the sciatic nerve in mice was established, followed by a single injection of bupivacaine, CU, or CUB around the sciatic nerve. Mechanical allodynia was assessed using a series of von Frey monofilaments applied to the plantar surface of the ipsilateral hind paw, while thermal hyperalgesia was assessed using the Hargreaves test. Both tests were performed at various time points according to the flowchart (**Figure** **4**a,b) to evaluate pain behavior up to 36 h post‐treatment, clarifying the applicability of the analgesic drug delivery system. Mechanical pain and thermal pain at different time points (up to 36 h after treatment) after the CCI model were tested to clarify the applicability of the as‐established analgesic drug delivery system.

Preliminary results showed that pain levels in the CCI model stabilized ≈28 days post‐surgery (Figure S8, Supporting Information). At 14 days post‐surgery, the sciatic nerve was blocked with a single administration of bupivacaine, CU, or CUB. The results demonstrated that the analgesic effect of CUB remained to be effective 24 h post‐injection. The paw withdrawal threshold (PWT) and paw withdrawal latency (PWL) of the CCI+CUB group significantly increased compared to the CCI group within 36 h. In contrast, the PWT and PWL in the CCI+Bupi group significantly increased within 6 h but showed a weaker analgesic effect than the CCI+CUB group (Figure 4c,d). Moreover, the analgesic effect of CUB on mechanical allodynia was more pronounced than on thermal hyperalgesia. In a word, the CUB treatment group produced a prolonged analgesic effect in CCI model mice compared to bupivacaine, with a more significant impact on mechanical allodynia than thermal hyperalgesia.

Clinically, diminished or lost mobility is a critical comorbidity in patients with peripheral neuropathic pain, leading to functional impairment and reduced quality of life. To evaluate whether CUB could improve locomotor function, we assessed movement activity in the open field test (OFT) (Figure 4e). Notably, mice treated with CUB exhibited the most motor traces in the OFT (Figure 4f). Neurological evaluations were performed daily using the Tarlov five‐point scoring system, with CUB‐treated animals showing better neurological outcomes than the other treatment groups (Figure 4g). Mice treated with CUB exhibited greater total distance traveled and higher average speed (Figure 4h,i). Additionally, the CUB‐treated mice showed improvements in both central movement distance and speed, indicating a reduction in pain. These behavioral changes suggest potential alleviation of cognitive dysfunction, including symptoms of depression and anxiety (Figure 4j,k).

Catwalk gait analysis further confirmed pain relief and locomotor function recovery in the CCI mice, as demonstrated by improvements in key parameters, including the pressure‐time image, thermal image, and gait patterns (Figure 4l–r). Computerized automated gait analysis (AGA) is employed to assess locomotor function after peripheral nerve injuries and to evaluate the effectiveness of experimental treatments for these injuries. The AGA system includes a glass walkway and a light source that illuminates the rodent's paw prints, which are then correlated with the pressure exerted by the paws. The functional recovery of the left hind limb was regularly monitored by calculating the sciatic functional index (SFI) scores of the mice. A score closer to zero indicates a favorable recovery of motor function. The wet weight ratio of the affected and intact gastrocnemius muscles also demonstrated a response to the recovery process from injury. Consistent with TEM images of a transverse section of sciatic nerve, these results indicated that the CUB group showed the best recovery of neuromotor function compared to the other three groups (Figure S9b–d, Supporting Information).

As shown in Figure 4m–r, compared to the CCI group, footprint analysis of CUB revealed significant decreases in swing phase, step cycle, and stride length, alongside significant increases in stance phase, duty cycle, and mean paw print intensity. Interestingly, the stride width in the CUB treatment group was slightly smaller than in the naive group, possibly due to incomplete recovery of muscle strength, leading to outward hind limb rotation. Automated gait analysis further indicated that the CUB group exhibited similar footprints to the naive group, particularly in terms of maximum contact intensity and contact area of the left hind limb (Figure 4l; Figures S10 and S11, Supporting Information). Moreover, the CUB treatment group displayed more regular gait patterns compared to the injury group (Figure S12, Supporting Information), consistent with footprint analysis results.

In vivo biosafety testing and biodistribution of CUB after local injection were performed. No apparent body weight loss was observed up to 28 days after injection of CUB at a dose of 20 ppm (Figure S9a, Supporting Information). When compared to the control group, no significant tissue damage or inflammatory response was observed in the sciatic nerve 28 days following the various treatments. In addition, we evaluated the potential toxicity of CUBs in mice. At 28 days post‐treatment, mice were euthanized, and blood samples, along with major organs, were collected for analysis. Serum levels of ALT, AST, ALP, urea, creatinine, and UA did not exhibit any notable differences between the groups (Figure S13, Supporting Information). Furthermore, no signs of necrosis, congestion, or bleeding were detected in the major organs (Figure S14, Supporting Information). Hemolytic assays confirmed that CUB did not induce hemolysis at the safe dosages (Figure S15, Supporting Information). Taken together, these findings suggest that CUB demonstrates excellent safety and does not cause toxicity in either healthy or CCI mice. Neuroglial cells surrounding axons form myelin sheaths and nerve membranes that support nerve fiber regeneration and repair by providing nutrients and promoting axon growth and maturation. After sciatic nerve injury, myocyte diameter and cross‐sectional area progressively decrease due to denervation, a reliable indicator of muscle atrophy. The CCI, Bupi, and CU groups displayed irregular nerve arrangement and poor nerve fiber continuity, while the CUB group demonstrated better axonal continuity, neatly arranged nerve fibers, more connective tissue between nerve bundles, neovascularization, regeneration of myelin sheaths, and a reduction in inflammatory cells (Figure S16a, Supporting Information). Measurements of gastrocnemius muscle fiber diameter and the number of myelin sheaths indicated superior nerve repair in the CUB group compared to other treatment groups (Figure S16b,c, Supporting Information). To assess the long‐term safety of CUB, we performed HE staining of the sciatic nerve and major organs, and Masson's trichrome staining of the gastrocnemius muscle after 12 weeks. The results showed no histopathological abnormalities, indicating that CUB does not induce nerve or muscle toxicity. Additionally, biochemical analyses of serum markers confirmed no systemic toxicity (Figures S17 and S18, Supporting Information). Altogether, these results suggest that CUB effectively accelerated motor function recovery in mice with CCI.

### 2.4 Balance Between Neuroimmune Signaling and Nerve Regeneration

Calcitonin gene‐related peptide (CGRP) is a neuropeptide consisting of 37 amino acids, primarily synthesized in the dorsal root ganglion (DRG) and the ventral horn of the spinal cord. This peptide plays a significant role in sensory signaling, as it is transported along the axons of sensory neurons to their terminals, both in the peripheral and central nervous systems. The highest concentrations of CGRP are found in the spinal cord, where it is involved in various physiological processes, including the modulation of pain and neuroinflammation.^[^ ^24^, ^25^, ^26^ ^]^ CGRP plays a critical and well‐established role in tissue repair, where its activation of the cognate G protein‐coupled receptor triggers intracellular signaling. Following tissue injury, elevated CGRP levels contribute to vasodilation, sensory transmission, immune modulation, and nerve regeneration.^[^ ^27^ ^]^ Additionally, CGRP plays a role in local neurogenic inflammation and nociception.^[^ ^28^ ^]^ Furthermore, the functions of immune cells, including macrophages, Langerhans cells, and T cells, are regulated by CGRP. Notably, thrombospondin‐1 (Thbs1), which encodes TSP‐1, a multifunctional extracellular matrix protein involved in tissue healing, is identified as the most significantly upregulated gene in both neutrophils and macrophages.^[^ ^29^ ^]^ Generally speaking, CGRP exerts immunomodulatory effects by promoting the autocrine secretion of TSP‐1.

**Figure 5:**
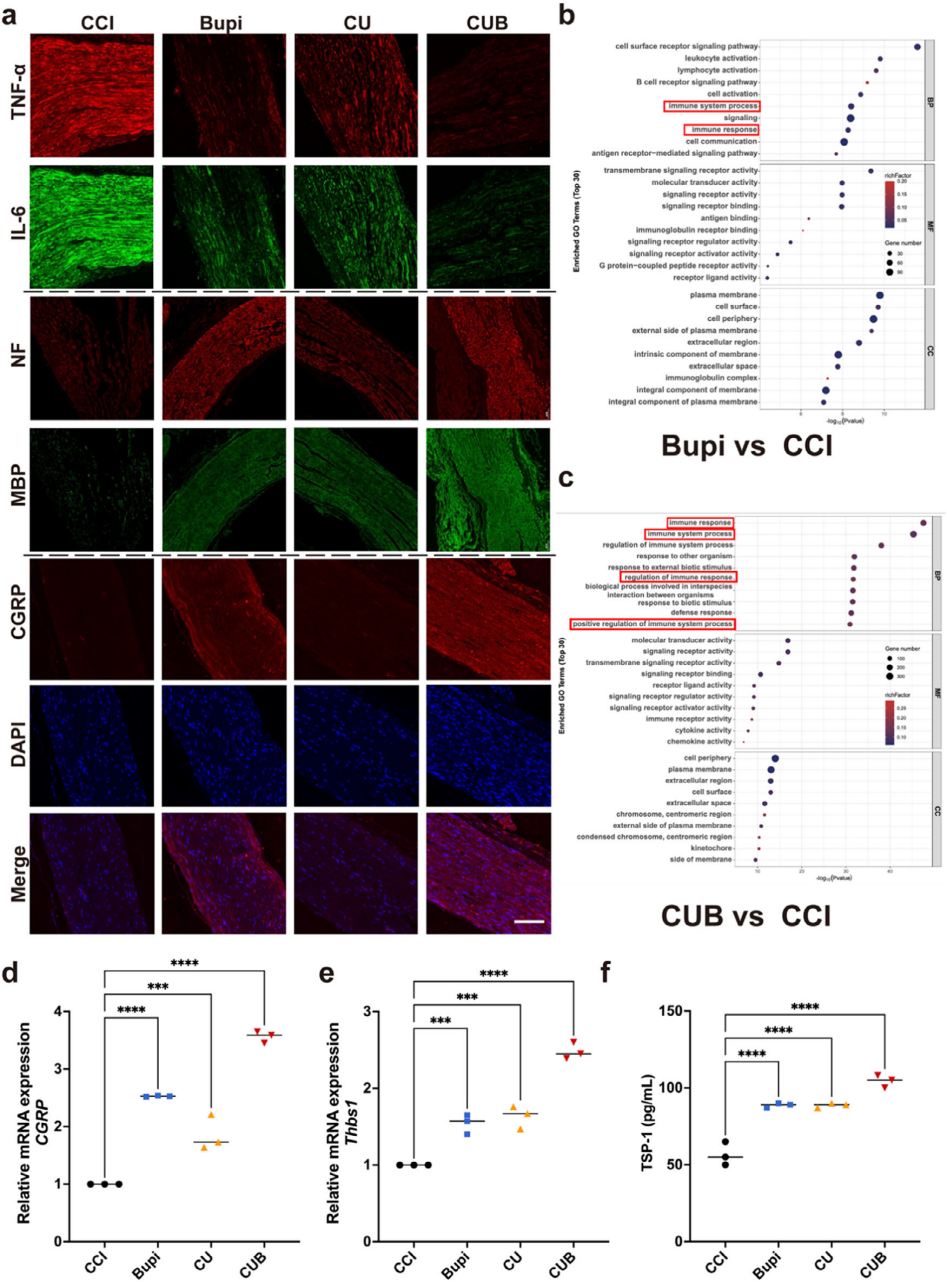
Nerve regeneration and neuroimmune signaling. a) Representative immunofluorescence of TNF‐α, IL‐6, IL‐10, NF, MBP, and CGRP in sciatic nerve 72 h after the different treatments of CCI mice. b,c) The KEGG pathway enriched upregulated genes for CCI mice with the Bupi‐treated group and the CUB‐treated group. d,e) Relative mRNA expression of CGRP and Thbs1 in sciatic nerve. f) Elisa for the determination of the concentration of TSP‐1 in the sciatic nerve. All data are expressed as the mean ± s.e.m. *P < 0.05; **P < 0.01; ***P < 0.001, ****P < 0.0001.

Staining of longitudinal sciatic nerve sections for inflammatory markers (TNF‐α, IL‐6) revealed that the Bupi and CUB groups showed enhanced anti‐inflammatory properties (**Figure** **5**a). Myelin regeneration indices (e.g., NF, MBP) were also assessed, showing that all three treatment groups exhibited significant differences from the CCI group, among which the CUB group had the strongest staining fluorescence intensity, indicating the best nerve regeneration ability (Figure 5a). Moreover, the CUB group demonstrated the most significant capacity to facilitate nerve repair and regeneration, as indicated by CGRP staining (Figure 5a). The relative expression of CGRP mRNA and fluorescence intensity in the sciatic nerve of different treatment groups showed consistent results (Figure 5d). Since Thbs1 encodes TSP‐1 to regulate biological processes, including tissue healing, we measured it separately. The results showed that the expression levels of different treatment groups were basically consistent with the expression levels of CGRP (Figure 5e,f). The CUB group showed the best TSP‐1 concentration, indicating that it has the strongest neural recovery ability.

As shown in Figure 5b,c, Gene Ontology (GO) and Kyoto Encyclopedia of Genes and Genomes (KEGG) Pathway Upregulated Enrichment Analysis revealed elevated signaling pathways with similarities between the two comparison groups. In mice treated with Bupi, the top‐ranking elevated pathways were predominantly associated with immune response processes, including immune system processes, cell surface receptor signaling, molecular transducer activity, and receptor signaling activity. These pathways were similarly significantly increased in the CUB group compared to the CCI group, along with increased regulation of the immune response and positive regulation of the immune system response. These findings suggest that both Bupi and CUB treatments facilitate nerve repair, primarily through the modulation of the immune response.

### 2.5 Intervening Neuropathic Pain Stimulation on Neuroglia with CUB In Vivo

**Figure 6:**
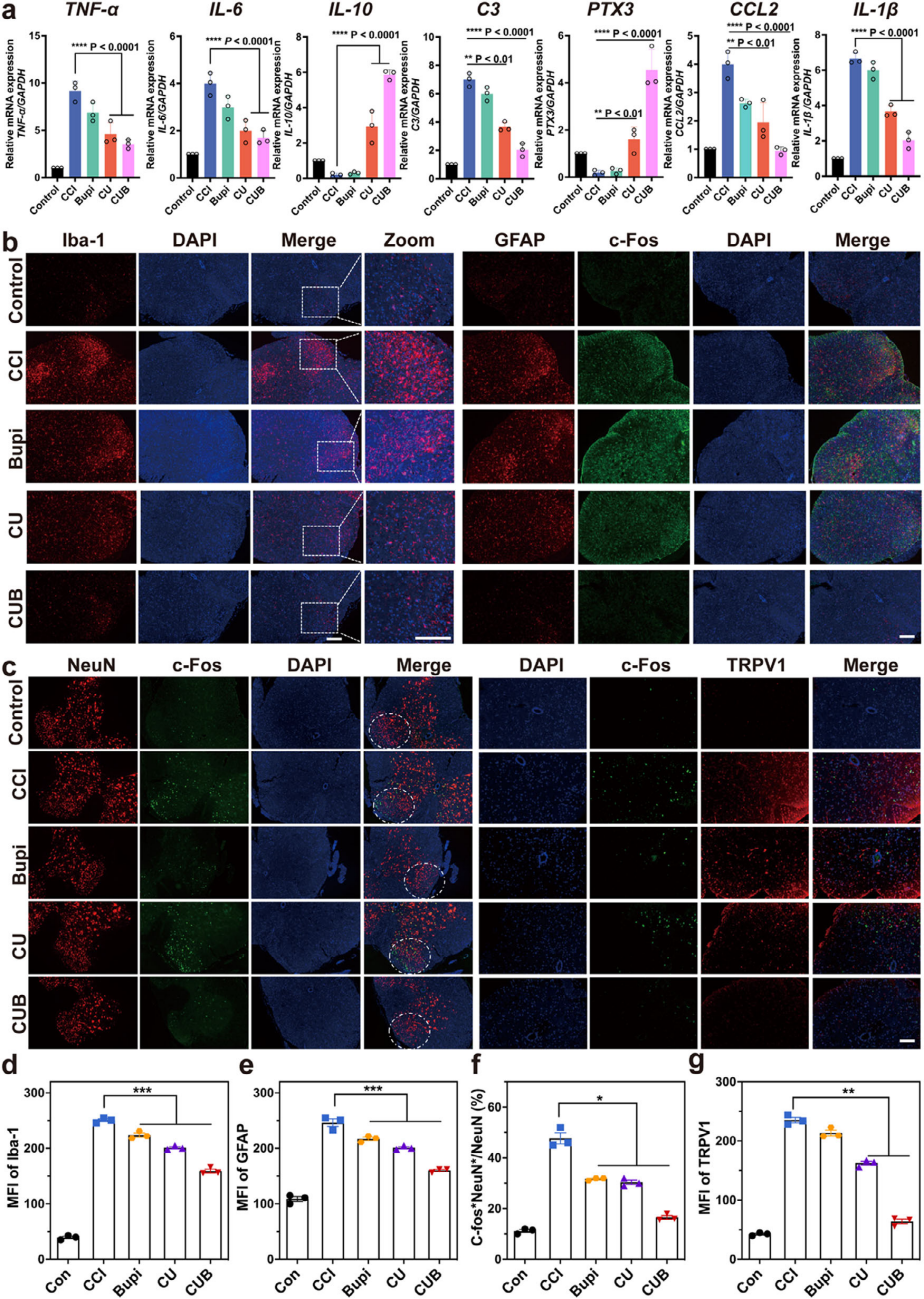
a) Relative mRNA expression of TNF‐α, IL‐6, IL‐10, C3, PTX3, CCL2and IL‐1β. Data are mean ± s.e.m. (n = 3). b) Representative immunofluorescence of Iba‐1, GFAP, and c‐Fos in the SG area of spinal cord dorsal horn neurons at 72 h after the different treatments of CCI mice. c) Representative immunofluorescence images of c‐Fos and NeuN in the SG area of spinal cord dorsal horn neurons were taken at 72 h after different treatments in CCI mice. Green indicates c‐Fos+ cells, red indicates NeuN+ cells, and blue represents DAPI+ (nuclear stain) cells. The merged image shows co‐localization of c‐Fos and NeuN. Additionally, immunofluorescence for TRPV1 in the spinal cord dorsal horn was performed, where green represents c‐Fos+, red represents TRPV1+, and blue represents DAPI+. The merged image highlights the co‐localization of c‐Fos and TRPV1. d–g) Statistical analysis of the proportion of c‐Fos positive neurons in NeuN neurons and mean fluorescence intensity (MFI) of Iba‐1, GFAP, and TRPV1. All data are expressed as the mean ± s.e.m. *P < 0.05, **P < 0.01, ***P < 0.001, ****P < 0.0001. All scale bars = 100 µm.

The spinal dorsal horn and neuroglia are key in pain transmission, neuroinflammation, and immune response. Nociceptors are triggered by damage‐associated molecular patterns (DAMPs) or pathogen‐associated molecular patterns (PAMPs), which are generated by invading pathogens or injured host cells, leading to the induction of pain, itch, or analgesia.^[^ ^30^ ^]^ Primary sensory neurons in the DRG transmit these signals to the spinal cord and CNS for pain perception. Microglia, key immune cells in the nervous system, provide support, protection, and waste removal. Astrocytes, abundant in the CNS, maintain homeostasis through neurotransmitter recycling, ion balance, synaptic modulation, and blood‐brain barrier integrity.^[^ ^31^ ^]^ Microglial polarization, influenced by transcription factors and signaling pathways, leads to either pro‐inflammatory M1‐like or anti‐inflammatory M2‐like phenotypes. Astrocytes also adopt A1‐like (pro‐inflammatory) or A2‐like (anti‐inflammatory) phenotypes during pain transmission.^[^ ^32^ ^]^ As shown in **Figure** **6**a, in the CUB treatment group, expression levels of TNF‐α and IL‐6, biomarkers of M1 microglia, were significantly inhibited compared to the CCI group, while the expression of IL‐10 and CD206, biomarkers of M2 microglia, was notably increased in both the CUB and CU groups compared to other treatment groups. Additionally, CUB and CU treatments led to statistically significant changes in the expression of C3, S100A10, and PTX3, biomarkers of A1 and A2 astrocytes, respectively. The levels of pro‐inflammatory factors, such as IL‐1β and CCL2 released upon activation, were also reduced in the CUB and CU groups (Figure 6a; Figure S19, Supporting Information). This result suggests that the prolonged analgesic effect of CUB promotes neuroglial polarization toward neuroprotection rather than neurotoxicity.

Pain‐related behavior tests combined with various neurobiological methods, such as immunofluorescence, further affirmed the satisfactory and long‐lasting analgesic effect of the CUB. To investigate neuronal activation, we examined spinal cord segments from the lumbosacral enlargement 72 h after different treatments. Immunofluorescence staining was performed to label microglia and astrocytes using Iba‐1 and GFAP markers, respectively. As shown in Figure 6b, compared to the control group, microglia and astrocytes in the spinal dorsal horn of CCI model mice were significantly activated. The single transient analgesic effect of Bupi was insufficient to suppress the activation of microglia and astrocytes, while CUB analgesia significantly inhibited neuroglial activation. The CU group also demonstrated a better effect to reduce neuroglial activation as compared to the Bupi group, suggesting that CU exhibits effective ROS‐scavenging activity, which alleviates neuroglial activation (Figure 6b).

Neurons are believed to have a crucial function in transmitting information within the nervous system, as they can be stimulated to produce conducting action potentials. Previous research has demonstrated that the neurons excitability in the substantia gelatinosa (SG) area of the spinal dorsal horn increases in the presence of pain, and that this hyperexcitability mediates the onset and development of pain.^[^ ^33^, ^34^ ^]^ In fact, the neuron‐glial cell signaling network within the spinal cord is essential for mediating information transmission between the peripheral and central nervous systems, especially in the process of noxious stimuli.^[^ ^35^ ^]^ NeuN and c‐Fos co‐staining of the SG area reflected the activation of positive neurons. The TRPV1 channel, part of the transient receptor potential cation channel subfamily V, is essential for transmitting nociceptive sensations. It is primarily involved in detecting noxious stimuli such as heat, acid, and various chemical irritants, and plays a pivotal role in the upward transmission of pain signals to the central nervous system. Therefore, the NeuN and c‐Fos co‐staining and TRPV1 channel staining of the spinal cord dorsal horn from CCI mice with different treatments were performed. As shown in Figure 6c, the control group showed minimal neuron activation in the spinal dorsal horn SG area, whereas the CCI group exhibited a significant increase in activated neurons. The CUB group showed the greatest inhibition of motor neurons, followed by the CU group, and finally the Bupi group (Figure 6c; Figure S20, Supporting Information). Statistical analysis revealed that both the proportion of c‐Fos positive neurons within NeuN‐expressing neurons and the mean fluorescence intensity (MFI) of Iba‐1, GFAP, and TRPV1 were significantly decreased in the CUB group compared to the CCI group (Figure 6d–g). These findings indicate that CUB treatment reduces pain by suppressing the activation of pain‐related neurons in the SG area of the spinal dorsal horn in vivo.

## 3 Conclusion

Chronic nerve injury and impaired regeneration remain significant challenges in the field of neurobiology, with limited effective treatments available. In this study, we proposed a new strategy by utilizing CUB with anti‐inflammatory and neuro‐regenerative properties for the treatment of chronic nerve injury. It has been demonstrated that the designed CUB could effectively mitigate neuroinflammation, promotes myelin regeneration, and enhances nerve repair through immune modulation, as evidenced by comprehensive histological, molecular, and functional assays. Specifically, the CUB significantly elevated TSP‐1 expression and activated CGRP signaling pathways, both of which are crucial for neural recovery. These findings not only highlight the potential of CUB as a therapeutic agent for nerve repair but also provide insight into the underlying mechanisms by which immune modulation can be harnessed to improve neural regeneration. Ultimately, this study offers promising new perspectives on chronic pain management and neural repair, contributing to the development of more effective treatments for nerve injuries.

## Conflict of Interest

The authors declare no conflict of interest.

## Author Contributions

Y.W. and X.J. contributed equally to this work. Y.W. and X.J. performed in methodology, validation, formal analysis, and resources, and wrote the original draft. Y.S. performed in investigation, resources, supervision, and funding acquisition. H.W. performed in formal analysis, writing‐review and editing. T.W. performed in methodology, validation, and resources. T.L. performed in methodology, validation. Y.C. performed in methodology, validation, and funding acquisition. J.Y. performed in resources, supervision, and funding acquisition. D.N. performed in investigation, resources, supervision, writing‐review and editing, funding acquisition, and project administration. H.J. performed in supervision, funding acquisition, and project administration.

## Supporting information



Supporting Information

## Data Availability

The data that support the findings of this study are available from the corresponding author upon reasonable request.

## References

[1] K. Inoue , M. Tsuda , Nat. Rev. Neurosci. 2018, 19, 138. 29416128 10.1038/nrn.2018.2

[2] S. P. Cohen , J. Mao , BMJ 2014, 348, f7656. 24500412 10.1136/bmj.f7656

[3] L. Colloca , T. Ludman , D. Bouhassira , R. Baron , A. H. Dickenson , D. Yarnitsky , R. Freeman , A. Truini , N. Attal , N. B. Finnerup , C. Eccleston , E. Kalso , D. L. Bennett , R. H. Dworkin , S. N. Raja , Nat. Rev. Dis. Primers 2017, 3, 17002. 28205574 10.1038/nrdp.2017.2 PMC5371025

[4] L. L. Mazaleuskaya , V. R. Muzykantov , G. A. FitzGerald , Trends Pharmacol. Sci. 2021, 42, 527. 33883067 10.1016/j.tips.2021.03.007 PMC8195851

[5] A. da Silva , S. Lepetre‐Mouelhi , P. Couvreur , Adv. Drug Delivery Rev. 2022, 187, 114359. 10.1016/j.addr.2022.114359 35654211

[6] F. A. Pinho‐Ribeiro , W. A. Verri , I. M. Chiu , Trends Immunol. 2017, 38, 5. 27793571 10.1016/j.it.2016.10.001 PMC5205568

[7] F. A. Pinho‐Ribeiro , L. Deng , D. V. Neel , O. Erdogan , H. Basu , D. Yang , S. Choi , A. J. Walker , S. Carneiro‐Nascimento , K. He , G. Wu , B. Stevens , K. S. Doran , D. Levy , I. M. Chiu , Nature 2023, 615, 472. 36859544 10.1038/s41586-023-05753-x PMC10593113

[8] H. Sies , V. V. Belousov , N. S. Chandel , M. J. Davies , D. P. Jones , G. E. Mann , M. P. Murphy , M. Yamamoto , C. Winterbourn , Nat. Rev. Mol. Cell Biol. 2022, 23, 499. 35190722 10.1038/s41580-022-00456-z

[9] J. Rosner , D. C. de Andrade , K. D. Davis , S. M. Gustin , J. L. K. Kramer , R. P. Seal , N. B. Finnerup , Nat. Rev. Dis. Primers 2023, 9, 73. 38129427 10.1038/s41572-023-00484-9 PMC11329872

[10] M. S. Woo , J. B. Engler , M. A. Friese , Nat. Rev. Neurosci. 2024, 25, 493. 38789516 10.1038/s41583-024-00823-z

[11] C. Chu , D. Artis , I. M. Chiu , Immunity 2020, 52, 464. 32187517 10.1016/j.immuni.2020.02.017 PMC10710744

[12] X. Hu , L. Du , S. Liu , Z. Lan , K. Zang , J. Feng , Y. Zhao , X. Yang , Z. Xie , P. L. Wang , A. M. Ver Heul , L. Chen , V. K. Samineni , Y.‐Q. Wang , K. J. Lavine , R. W. Gereau , G. F. Wu , H. Hu , J. Clin. Invest. 2023, 133, 5. 10.1172/JCI161507 PMC9974096 36701202

[13] S. Talbot , S. L. Foster , C. J. Woolf , Annu. Rev. Immunol. 2016, 34, 421. 26907213 10.1146/annurev-immunol-041015-055340

[14] M. Enamorado , W. Kulalert , S.‐J. Han , I. Rao , J. Delaleu , V. M. Link , D. Yong , M. Smelkinson , L. Gil , S. Nakajima , J. L. Linehan , N. Bouladoux , J. Wlaschin , J. Kabat , O. Kamenyeva , L. Deng , I. Gribonika , A. T. Chesler , I. M. Chiu , C. E. Le Pichon , Y. Belkaid , Cell 2023, 607, 186. 36640762 10.1016/j.cell.2022.12.037 PMC11512587

[15] P. Hanč , M.‐A. Messou , J. Ajit , U. H. von Andrian , Trends Immunol. 2024, 45, 783. 39307581 10.1016/j.it.2024.08.007 PMC11493364

[16] D. Qian , J. Xu , X. Zhang , F. Hu , S. Cao , Y. Dong , X. Liu , Y. Yao , H. Yu , Y. Lu , X. Ma , K. Cheng , X. Zhao , G. Nie , X. Zhang , Adv. Mater. 2024, 36, 2307624. 10.1002/adma.202307624 39478649

[17] T. Wu , G. Chen , J. Han , R. Sun , B. Zhao , G. Zhong , Y. Yamauchi , B. Guan , J. Am. Chem. Soc. 2023, 145, 16498. 37477359 10.1021/jacs.3c03029

[18] Z. Zhou , M. Vázquez‐González , I. Willner , Chem. Soc. Rev. 2021, 50, 4541. 33625421 10.1039/d0cs01030h

[19] P. Gao , Y. Chen , W. Pan , N. Li , Z. Liu , B. Tang , Angew. Chem., Int. Ed. 2021, 60, 16763. 10.1002/anie.202102574 33686725

[20] H. Wang , J. C. Hsu , W. Song , X. Lan , W. Cai , D. Ni , Natl. Sci. Rev. 2024, 11, nwae280. 39257435 10.1093/nsr/nwae280 PMC11384914

[21] R. Ettlinger , U. Lächelt , R. Gref , P. Horcajada , T. Lammers , C. Serre , P. Couvreur , R. E. Morris , S. Wuttke , Chem. Soc. Rev. 2022, 51, 464. 34985082 10.1039/d1cs00918d

[22] H. Ma , Z. Pan , B. Lai , C. Zan , H. Liu , Drug Des., Dev. Ther. 2023, 17, 2639. 10.2147/DDDT.S417051 PMC10475288 37667787

[23] X. Ji , J. Zhou , Z. Zhou , Z. Liu , L. Yan , Y. Li , H. Guo , W. Su , H. Wang , D. Ni , Bioact. Mater. 2024, 42, 112. 39280583 10.1016/j.bioactmat.2024.08.024 PMC11402068

[24] Y. Zhang , J. Yang , P. Zhang , T. Liu , J. Xu , Z. Fan , Y. Shen , W. Li , H. Zhang , Sci. Rep. 2016, 6, 27724. 27296555 10.1038/srep27724 PMC4906351

[25] J. Larouche , S. Sheoran , K. Maruyama , M. M. Martino , Adv. Wound Care 2018, 7, 209. 10.1089/wound.2017.0761 PMC6032665 29984112

[26] P. J. Muire , L. H. Mangum , J. C. Wenke , Front. Immunol. 2020, 11, 1056. 32582170 10.3389/fimmu.2020.01056 PMC7287024

[27] K. Tsujikawa , K. Yayama , T. Hayashi , H. Matsushita , T. Yamaguchi , T. Shigeno , Y. Ogitani , M. Hirayama , T. Kato , S.‐I. Fukada , S. Takatori , H. Kawasaki , H. Okamoto , M. Ikawa , M. Okabe , H. Yamamoto , Physiology 2007, 104, 16702. 10.1073/pnas.0705974104 PMC2034234 17923674

[28] A.‐M. Salmon , M. I. Damaj , L. M. Marubio , M. P. Epping‐Jordan , E. Merlo‐Pich , J.‐P. Changeux , Nat. Neurosci. 2001, 4, 357. 11276224 10.1038/86001

[29] Y.‐Z. Lu , B. Nayer , S. K. Singh , Y. K. Alshoubaki , E. Yuan , A. J. Park , K. Maruyama , S. Akira , M. M. Martino , Nature 2024, 628, 604. 38538784 10.1038/s41586-024-07237-y PMC11023938

[30] C. R. Donnelly , C. Jiang , A. S. Andriessen , K. Wang , Z. Wang , H. Ding , J. Zhao , X. Luo , M. S. Lee , Y. L. Lei , W. Maixner , M.‐C. Ko , R.‐R. Ji , Nature 2021, 591, 275. 33442058 10.1038/s41586-020-03151-1 PMC7977781

[31] H.‐G. Lee , M. A. Wheeler , F. J. Quintana , Nat. Rev. Drug Discovery 2022, 21, 339. 35173313 10.1038/s41573-022-00390-x PMC9081171

[32] X. Lan , X. Han , Q. Li , Q.‐W. Yang , J. Wang , Nat. Rev. Neurol. 2017, 13, 420. 28524175 10.1038/nrneurol.2017.69 PMC5575938

[33] N. T. Fiore , S. R. Debs , J. P. Hayes , S. S. Duffy , G. Moalem‐Taylor , Nat. Rev. Neurol. 2023, 19, 199. 36859719 10.1038/s41582-023-00777-3

[34] S. Yin , P. Gao , L. Yu , L. Zhu , W. Yu , Y. Chen , L. Yang , Adv. Sci. 2022, 9, 2202735. 10.1002/advs.202202735 PMC9443434 35750652

[35] V. Gangadharan , H. Zheng , F. J. Taberner , J. Landry , T. A. Nees , J. Pistolic , N. Agarwal , D. Männich , V. Benes , M. Helmstaedter , B. Ommer , S. G. Lechner , T. Kuner , R. Kuner , Nature 2022, 606, 137. 35614217 10.1038/s41586-022-04777-z PMC9159955

