# Supplementary material for: Nano‐Anesthetics Regulate Neuro‐Immune Interaction for Treating Neuropathic Pain
Source: Adv Sci (Weinh). 2025 May 21;12(29):e02920. doi: 10.1002/advs.202502920 (PMC12362777; doi:10.1002/advs.202502920)
Supplement: Supplementary file 1 — Supporting Information [file ADVS-12-e02920-s001.docx]

Supporting Information

**Nano-anesthetics Regulate Neuro-Immune Interaction for Treating Neuropathic Pain**

Yue Wang ^a#^, Xiuru Ji ^b#^, Yu Sun ^a^**^*^**, Han Wang ^b^, Ting Wang ^a^, Tao Luo ^a^, Yanyong Cheng ^a^, Jia Yan ^a^, Dalong Ni ^b,c^**^*^** and Hong Jiang ^a^**^*^**

^a^ Department of Anesthesiology, Shanghai Ninth People’s Hospital, Shanghai Jiao Tong University School of Medicine, Shanghai 200011, P.R China

^b^ Department of Orthopaedics, Shanghai Key Laboratory for Prevention and Treatment of Bone and Joint Diseases, Shanghai Institute of Traumatology and Orthopaedics, Ruijin Hospital, Shanghai Jiao Tong University School of Medicine, Shanghai 200025, P.R China

^c^ Department of Biomaterials and Stem Cells, Suzhou Institute of Biomedical Engineering and Technology, Chinese Academy of Science, Suzhou 215163, P. R. China

**^*^** Corresponding author

E-mail: suny1333@sh9hospital.org.cn (Y. Sun), ndl12353@rjh.com.cn (D. Ni), jiangh1173@sh9hospital.org.cn (H. Jiang)

Yue Wang and Xiuru Ji contributed equally to this work.

**Experimental Section**

**Materials**

**Synthesis of Ce-UIO-66-Bupivacaine (CUB)**

For the synthesis of Ce-UIO-66, 4 mmol (NH_4_)_2_Ce(NO_3_)_6_ was dissolved in H_2_O (4 mL) and added to a mixed solution consisting of fumaric acid (4 mmol) and PVP (50 mg) in DMF (30 mL) at a temperature of 100 ℃, followed by stirring for 15 min to obtain Ce-UIO-66 (CU). Subsequently, a solution of bupivacaine at a concentration of 40 μg/mL was added to the dissolved CU and stirred at 25 ℃ for 24 h, leading to the formation of Ce-UIO-66-Bupivacaine (CUB).

**Measuring instruments**

Transmission electron microscopy (TEM) images were captured with an FEI Talos F200X. X-ray diffraction (XRD) analyses were conducted using a Rigaku D/MAX-2250 V. Fourier transform infrared (FTIR) spectroscopy was performed on a Thermo Fisher Nicolet 6700. The Brunauer-Emmett-Teller (BET) surface area and pore size were accessed with a TriStar II 3020. Thermogravimetric analysis was conducted with the NETZSCH STA 449F3. Dynamic light scattering (DLS) was measured using a Malvern Zetasizer Nano S. Confocal laser scanning microscopy was conducted with a Leica TCS SP8 STED 3X.

**Isolation of Primary Mouse Spinal Cord Microglia and Astrocytes**

The spinal cords were extracted from neonatal (P0 to P3) C57BL/6J mice, and the dural membrane were carefully removed in ice-cold HBSS at 4°C. Following enzymatic dissociation with 0.25% trypsin for 10 minutes at 37°C, the tissue was washed twice with HBSS and gently triturated in a culture medium consisting of DMEM/F12 (Gibco, 11330032), 10% fetal bovine serum, and 1% penicillin-streptomycin solution. The resulting cell suspension was filtered through a 70-μm sieve and subsequently cultured in T75 flasks coated with poly-D-lysine (Gibco, A3890401), adding 10 mL of culture medium. The medium was replaced every 4-5 days until the cells approached confluence. After 18-22 days of culture, microglia were collected by gentle shaking for 30 minutes, followed by centrifugation for 5 minutes. The remaining cells, which were firmly attached to the flask surface, were astrocytes. The separated cell pellets were resuspended in culture medium and plated in confocal or 96-well plates for further experiments.

**Cell Culture**

All medium types were supplemented with 10% certified heat-inactivated fetal bovine serum (FBS, Gibco, U.S.A.), penicillin (100 U/mL), and streptomycin (100 mg/ml), and the cells were cultured at 37°C in a humidified 5% CO_2_ atmosphere.

**Measurement of Antioxidant Ability of CUB By Confocal Microscopy**

A 3 mL solution of H₂O₂ (100 μM) was mixed with different concentrations of CUB (1 mL, ranging from 0 to 40 μg/mL). The reaction was left to proceed for several minutes, after which it was allowed to reach completion. Subsequently, the remaining H₂O₂ concentration in each sample was measured using a hydrogen peroxide assay kit (Beyotime Biotechnology, S0038). The residual H₂O₂ concentration served as an indicator of the antioxidant capacity of CUB.

ROS levels were measured using fluorescence microscopy, employing the fluorescent probe DCFH-DA. Briefly, microglia and astrocytes were seeded in confocal dishes, each containing a glass slide (2 × 10⁵ cells/well), and cultured for 24 hours to allow cell adherence. After incubating the cells with H₂O₂ (100 μM) and lipopolysaccharide (LPS, 1 μg/mL) for 12 hours, the medium was replaced with Bupi, CU, or CUB, and cells were further incubated for an additional 12 hours. Following this, the cells were stained with 10 μM DCFH-DA (DOJIND, R253) and 10 μg/mL Hoechst 33342 (Invitrogen, H3570, U.S.A.) for 30 minutes at 37°C in the dark. The cells were washed three times with sterile PBS to remove any unbound DCFH-DA dye. The fluorescence intensity of DCF was quantified at an excitation wavelength of 488 nm, reflecting the intracellular ROS levels. The LPS, LPS+Bupi, LPS+CU, LPS+CUB treated microglia and astrocytes were fixed by 4% paraformaldehyde (PFA) for 15 min. After washed with PBS three times, the prepared membrane-breaking solution (3‰ Triton) was added into each well to break the membrane for 10 min. Primary antibody IL -6 (Proteintech, 66146-1-Ig)/IL-10 (Proteintech, 60269-1-Ig) was prepared according to the instructions and the ratio of the pre-test, 200 μL of which were added to each well, and put in the refrigerator at 4°C overnight. We then configured the secondary antibody according to the source of the primary antibody, and left for 2 h in the dark room. Hoechst working solution (diluted 1:1000 with PBS) was added to each well and placed in a dark room. The plates were finally sealed with 50% glycerol. The imaging process was conducted using the Opera Phenix Plus High-Content Screening system (PerkinElmer, U.S.A.), which is known for its advanced imaging capabilities.

**Enzyme-linked Immunosorbent Assay Analysis (ELISA)**

The mice were euthanized using CO2, after which the L4–L6 spinal cord segments and left sciatic nerves were promptly removed and homogenized in phosphate-buffered saline (PBS). The homogenized sample was subsequently centrifuged at 15,000 g for 60 minutes at 4 °C, and the supernatant obtained was collected for further analysis. ELISA kits for mouse TSP-1 (from Abclonal, RK03230) and CGRP (from Elabscience Biotechnology Co. Ltd, E-EL-M0215c) were used to measure the relevant proteins in the supernatant, following the manufacturer's guidelines.

**Animal Experiment**

Adult male C57BL/6 mice weighing between 20 and 30 g (Shanghai Jie Si Jie Experimental Animal Co., Ltd.) were used in the experiment. The study protocol was reviewed and approved by the Ethics Committee of Shanghai Jiao Tong University (A202124). All mice were maintained under a 12-hours light-dark cycle and provided ad libitum access to standard rodent chow and water.

**Chronic Constriction Injury (CCI) model**

After anesthetizing the mice with isoflurane, the left hind limb's sciatic nerve was carefully exposed. Using a 4-0 chromium enteric suture, four square knots were tied at 1 mm intervals along the main nerve trunk. The knots were tightened just enough to induce a mild tremor in the peripheral muscle group, while ensuring only light compression of the sciatic nerve to maintain blood flow. In the sham procedure, the sciatic nerve was exposed without any ligation.

**Von Frey Test**

Before testing, mice were acclimated to the pain testing apparatus, which was enclosed in a Plexiglas chamber, for 30 minutes. Signs of acclimation included quiet behavior and reduced voluntary movement. Calibrated von Frey filaments (with bending forces of 0.008, 0.4, 0.6, 1, 1.4, and 2 g) were applied vertically to the plantar surface near the incision site on the mouse’s paw until the filament bent. Each filament was applied five times with a minimum 1-minute interval between applications. Each stimulation lasted for 1 second, starting with the 0.008 g filament and progressively increasing to 2 g. Valid pain responses were identified based on pain-related behaviors, including foot withdrawal occurring three or more times. The mechanical pain threshold (PWT) was determined by the last filament value recorded. Baseline measurements were collected one day prior to the incision, and both the PWT and its variations were documented at designated time points for different mouse groups.

**Hargreaves Test**

In the two days prior to testing and on each test day, the animals were allowed to acclimate to both the testing environment and the equipment for 30 minutes. Each mouse was individually housed in a Plexiglas chamber, which was positioned on a raised glass surface, kept at a stable thermo-neutral temperature of 30°C. Thermal sensitivity was assessed by focusing a light beam from below onto the plantar surface of the hind paw. The time taken for the paw to withdraw from the heat source was recorded as the paw withdrawal latency (PWL), measured in seconds. The intensity of the light was adjusted to elicit typical PWL values of 10–14 seconds in control mice. To prevent tissue injury from overheating, the light beam automatically turned off after 20 seconds. If the hind paw did not withdraw within this time frame, a PWL of 20 seconds was assigned. For analysis, the average of two PWL measurements, taken at least 5 minutes apart for each hind paw, was used.

**Open Field Test and Tarlov Score**

This study examines spontaneous locomotor activity in mice using an open-field test paradigm. After surgery and drug treatments, mice were positioned at the center of a 40 × 40 × 60 cm open area and given the freedom to explore for a minimum of 30 minutes. The mice's movement paths and total distance traveled were monitored using cameras linked to a computerized video tracking system. During the first 5 minutes of exploration, the Tarlov score was assessed as follows.

Tarlov Score:

Score 0: Complete paralysis of the lower extremities.

Score 1: Detectable joint movements of the lower extremities.

Score 2: Lower limbs can move freely, but cannot stand.

Score 3: Can stand but cannot walk.

Score 4: Lower limb motor function is completely restored and can walk normally.

**Catwalk Gait Analysis**

To evaluate specific functional alterations in gait, gait analysis was conducted using the CatWalk method with the CatWalk™ XT system (Noldus Information Technology, Wageningen, The Netherlands). Mice were allowed to cross a transparent corridor, with the floor being observed by a charge-coupled device (CCD) camera placed beneath it. Fluorescent lighting illuminated the corridor, enabling the camera to capture variations in the gait of the left front (LF), right front (RF), left hind (LH), and right hind (RH) paws. The intensity of the light reflected the force exerted by each paw. Real-time footprint images were recorded and analyzed with the CatWalk XT software, from which pain-related gait parameters were calculated. In addition, quantitative analyses of key gait parameters were performed. Prior to surgery, animals under training on the CatWalk runway to complete continuous runs in order to minimize stress. The trial was strictly repeated until three uninterrupted runs were successfully recorded. Mice that showed prolonged stopping or reversed direction during the trial were deemed to have failed. No reinforcement, either positive or negative, was utilized to encourage voluntary walking. All trials and mechanical withdrawal threshold measurements were completed on the same day.

As described in previous reports, a technician, blinded to the experimental conditions, assessed the SFI (Sciatic Function Index) in the different animal groups on a weekly basis following surgery. Several key parameters were extracted from the footprint analysis, and all measurements were recorded for both the experimental and control groups. An SFI score of 0 indicated normal motor function, while a score of -100 represented a complete loss of motor function.

SFI=$118.9\times\frac{TSe-TSn}{TSn}-51.2\times\frac{PLe-PLn}{PLn}-7.5$

TS: toe spread, PL: print length, which were main parameters recorded by CatWalk™ XT.

**Immunofluorescence**

Mice were subjected to urethane anesthesia (1.7 g/kg, i.p.), followed by perfusion with phosphate-buffered saline (PBS), and their tissues were preserved using 4% paraformaldehyde (PFA). The spinal cords and sciatic nerves were promptly collected for sectioning. After an overnight post-fixation in 4% PFA, the tissues were dehydrated in 30% sucrose. Once the tissue sank to the bottom, it was sectioned at a thickness of 25 μm using a freezing microtome (Leica, Wetzlar, Germany). Sections were then washed three times for 5 minutes each with PBS containing 0.3% Triton X-100, followed by blocking with 1% BSA and 0.3% Triton X-100 in PBS for 2 hours. Next, the sections were incubated overnight at 4°C with primary antibodies: rabbit anti-c-Fos (1:1000, Cell Signaling Technology), mouse anti-NeuN (1:1000, Abcam), rabbit anti-IBA1 (1:1000, Abcam), rabbit anti-GFAP (1:1000, Abcam), mouse anti-TRPV1 (1:1000, Abcam), mouse anti-IL-6 (1:1000, Abcam), mouse anti-IL-10 (1:1000, Abcam), mouse anti-TNF-α (1:1000, Abcam), rabbit anti-NF (1:1000, Abcam), mouse anti-MBP (1:1000, Abcam), and mouse anti-CGRP (1:1000, Abcam). After washing with PBS containing 0.3% Triton X-100 (5 minutes, 3 times), sections were incubated for 2 hours at room temperature with secondary antibodies: goat anti-rabbit IgG H&L (1:1000, Abcam) and goat anti-mouse IgG H&L (1:1000, Abcam), protected from light. Ultimately, the sections were placed onto glass slides, and images were obtained using a fluorescence microscope (Olympus, Tokyo, Japan). The fluorescence intensity was measured using ImageJ (v.1.8 NIH, Washington, USA), and the proportion of fluorescence-positive cells was calculated. For quantification of c-Fos, NeuN, TRPV1, Iba1, and GFAP expression, images of the L4-L6 spinal cord were outlined in standardized regions of interest (ROIs) using ImageJ software. The fluorescence-positive area percentage within each ROI was determined. The total number of cells in each ROI was counted, and double-positive cells (yellow fluorescence) were manually identified and counted by a technician blinded to the experimental conditions and groupings.

**Western Blotting**

Tissues were minced using a liquid nitrogen frozen mincer, followed by centrifugation at 4°C for 10 min. The resulting tissue supernatants were collected and lysed using RIPA Lysis and Extraction Buffer (Thermo Fisher Scientific). Protein concentration was then measured using a Pierce BCA protein assay kit (A65453; Thermo Fisher Scientific). Equal amounts of protein were separated by SDS-PAGE and transferred to a nitrocellulose membrane (MilliporeSigma, Burlington, MA, USA). The membranes were then blocked with 5% milk in Tris-buffered saline with Tween 20 (TBST; ST828; Beyotime), followed by incubation with anti-p-ERK (Rabbit polyclonal, 1:1000, Cell Signaling Technology), anti-ERK (Rabbit monoclonal, 1:1000, Cell Signaling Technology), anti-Tubulin (Mouse monoclonal, 1:5000, Sigma) at 4°C overnight. After washing with TBST, membranes were incubated with HRP-conjugated secondary antibodies (anti-rabbit IgG and anti-mouse IgG, both at 1:5000) for 1 hour at room temperature. Blots were visualized using enhanced chemiluminescence (ECL, Thermo Scientific) and quantified using ImageJ software.

**Flow Cytometry Analysis:**

The treated microglia and astrocytes were digested with 0.25% trypsin and then centrifuged and resuspended. Anti-CD206 and anti-S100A10 antibodies were added respectively and incubated for 25 min with PBS. After washing with PBS, cells were collected by flow cytometry (FACS Calibur™, BD Biosciences) and analyzed by FlowJo 10.

**RNA Sequencing (RNA-seq) and Data Analysis**

Total RNA was isolated from the sciatic nerves using TRIzol Reagent (Invitrogen, USA). The integrity of the RNA was assessed with a 2100 Bioanalyzer (Agilent Technologies, USA), while its concentration was measured using a Qubit 2.0 fluorometer in combination with the Qubit RNA Assay Kit (Life Technologies, USA). Libraries for sequencing were prepared with the Illumina TruSeq RNA Sample Prep Kit (San Diego, USA), and sequencing was performed on an Illumina HiSeq 2500 platform (San Diego, USA). The mRNA expression levels of unigenes were determined using TopHat v2.0.9 and Cufflinks, with the data subsequently normalized to FPKM. Differential gene expression was identified according to specified criteria: FDR < 0.01, fold change <0.5 or >2.0 (log2 ratio <−1 or >1), and P value < 0.05.

**Quantitative Real-Time PCR**

CCI mice were treated with Bupi, CU, and CUB respectively. At 36 h after treatment, mice were euthanized and sciatic nerves and spinal cords were collected. The LPS, LPS+Bupi, LPS+CU, LPS+CUB treated microglia and astrocytes were seeded in 12-well plates at a density of 1×10^6^ cells per well. Total RNA was extracted from tissues or cells using Trizol Reagent, following the manufacturer's instructions. Subsequently, the RNA was reverse transcribed into complementary DNA (cDNA) using the First Strand cDNA Synthesis Kit (Takara, RR420A). Real-time PCR was carried out using the SYBR Green qPCR Master Mix (Takara, RR036A). The specific primers used for the PCR analysis are listed in Supplementary Table 1.

**Biosafety Assessment**

The cytotoxicity of Bupi, CU, and CUB was assessed using the Cell Counting Kit-8 (CCK-8) assay (DOJIN, CK04). Cells were seeded into 96-well plates and treated with 90 µL of medium and 10 µL of the test materials. After 72 hours, 10 µL of CCK-8 solution was added, and the cells were further incubated for 2 hours. Optical density (OD) values were measured at 450 nm using a microplate reader, and cell viability was subsequently calculated. To further evaluate the biocompatibility, the nanomaterial was co-cultured with microglia and astrocytes for 72 hours before performing nuclear staining and Live/Dead staining. Cell morphology was observed under an inverted fluorescence microscope. 2 v/v % mouse blood is first centrifuged at 2000 rpm for 5 minutes, then the centrifuged erythrocytes are resuspended in an equal volume of PBS (pH=7.4) and centrifuged again at 2000 rpm for 5 minutes, and the process was repeated until the supernatant was almost clear and colorless. A graded concentration of HPTA hydrogel was weighed into small centrifuge tubes and 1 mL of hematocrit (1 mL in PBS=7.4) was added to each tube and incubated for 2 h at 37°C in an incubator as the test sample. Following incubation, 100 μL of the supernatant from each sample was uniformly added to a 96-well plate, and the OD value was subsequently measured at 576 nm.

The major organs, including the skin, heart, liver, spleen, lungs, kidneys, and spinal cord, from mice in different treatment groups were harvested and stained with hematoxylin-eosin (H&E) for histological examination. Blood samples were also obtained and analyzed using an automatic hematology analyzer. The leftover blood underwent centrifugation at 3000 rpm for 15 minutes at 4°C to separate the serum. This serum was then tested for various biomarkers associated with liver and kidney function, including uric acid (UA), urea, creatinine (CRE), alanine aminotransferase (ALT), alkaline phosphatase (ALP) and aspartate aminotransferase (AST). Additionally, liver and kidney tissues were weighed, homogenized in PBS (ninefold volume), and centrifuged at 4000 rpm for 10 minutes at 4°C. The resulting supernatant was collected to quantify ALP levels. The resulting supernatant was collected to measure ALP levels. All biochemical parameters were assessed using an automated biochemical analyzer. In addition, liver and kidney tissues were weighed, homogenized in PBS (ninefold volume), and centrifuged at 4000 rpm for 10 minutes at 4°C. The resulting supernatant was collected to quantify ALP levels. All biochemical analyses were performed using an automated biochemical analyzer.

**Statistical Analysis**

Statistical analysis was performed using Prism 10.0 software (GraphPad, USA). All raw data and results were analyzed in a blinded manner. Quantitative data are presented as the mean ± standard error of the mean (s.e.m.). Differences between two groups were assessed using a two-tailed Student's t-test. For comparisons involving three or more groups, one-way ANOVA was used, followed by the Bonferroni post hoc test for multiple comparisons. Repeated measures ANOVA was applied to analyze data with multiple measurements over time. A P value of less than 0.05 was considered statistically significant, with asterisks denoting the level of significance (**P* < 0.05, **P *<* 0.01, ****P* < 0.001 and *****P* < 0.0001).

**Supplementary Figures**


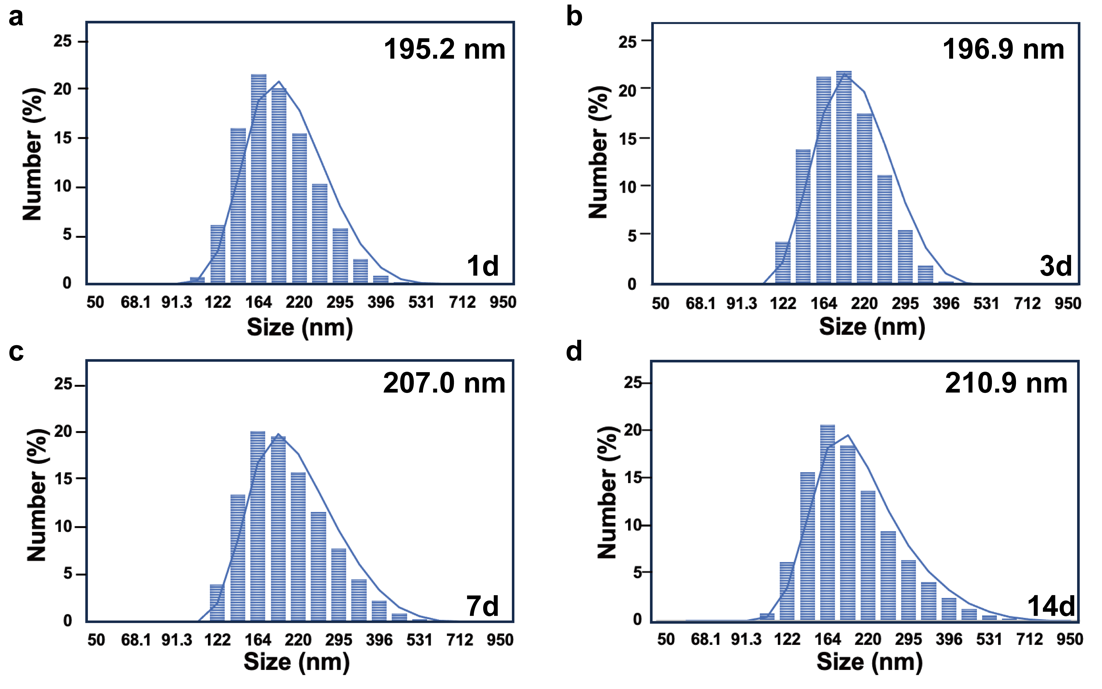


**Figure S1.** a-d) Nanoparticle size frequency distribution over multiple time points (1, 3, 7, and 14 days).


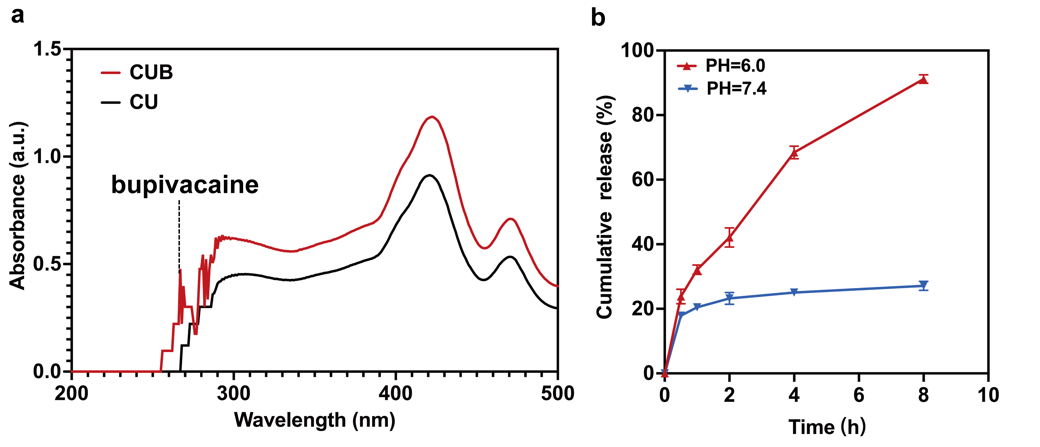


**Figure S2.** a) UV-Vis of CU and CUB. b) Cumulative release under simulated tissue injury conditions (pH=6.0) and physiological conditions (pH=7.4).


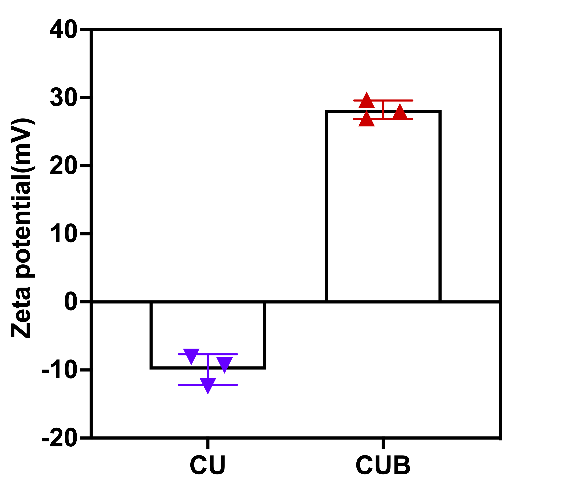


**Figure S3.** Zeta potential of CU and CUB.


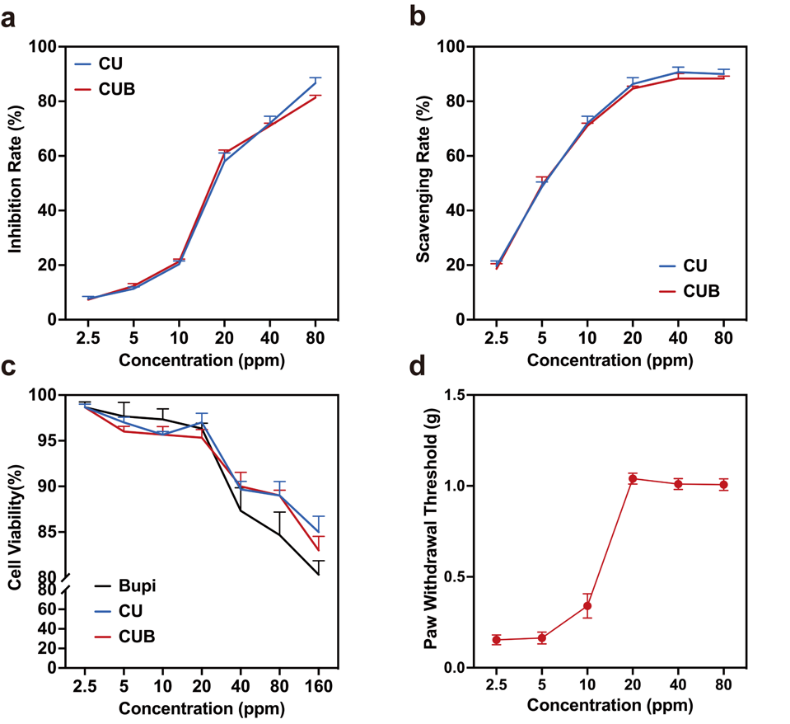


**Figure S4.** a) The total antioxidant capacity of CU and CUB. b) The ROS scavenging ability of CU and CUB. c) Cell viability of different concentrations of CUB on microglia. d) Mechanical paw withdrawal threshold of CCI mice treated with different concentrations of CUB (n = 3, mean ± SEM).


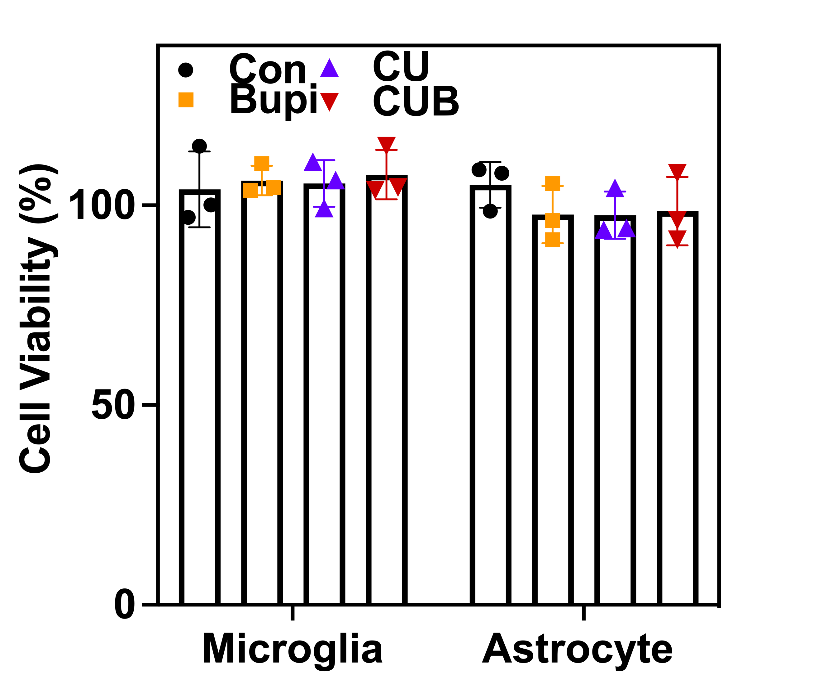


**Figure S5.** Cell viability of Bupi, CU and CUB on microglia and astrocyte after co-cultured for 72 h.


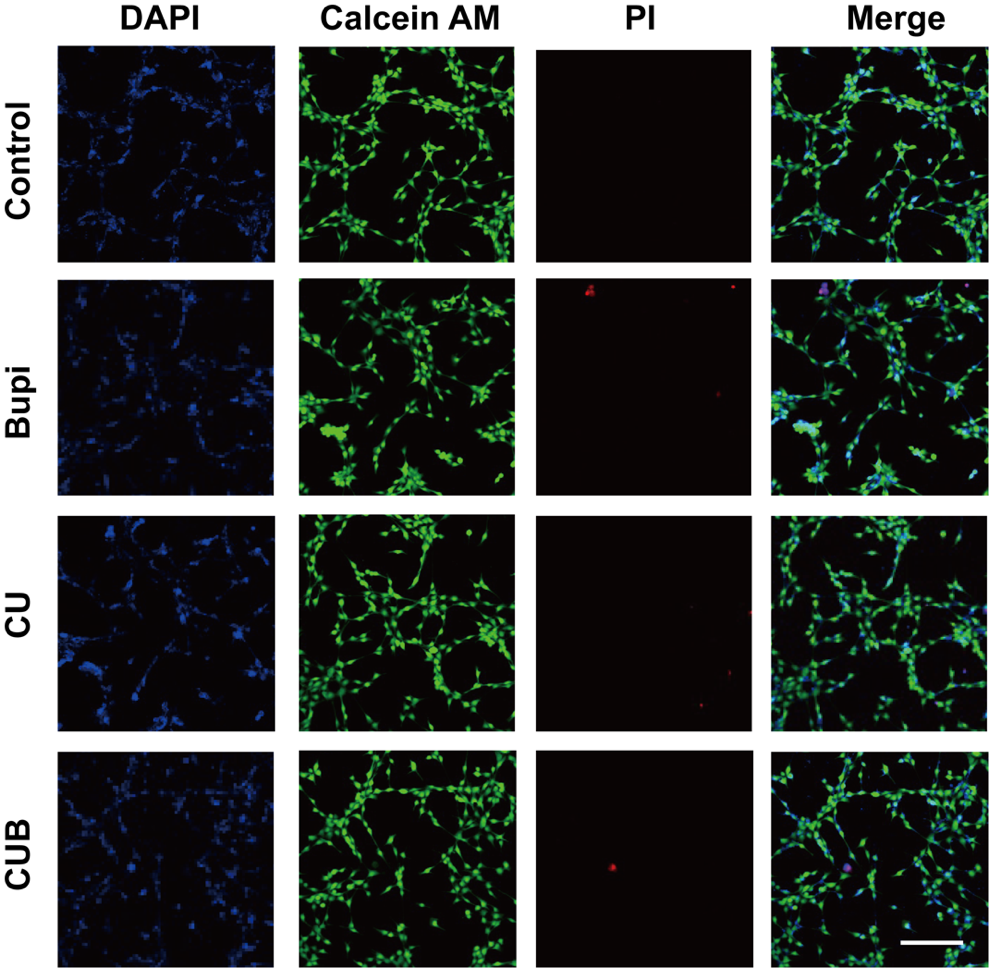


**Figure S6.** Live/dead staining of microglia after co-cultured for 72 h. Scale bar = 100 μm.

**
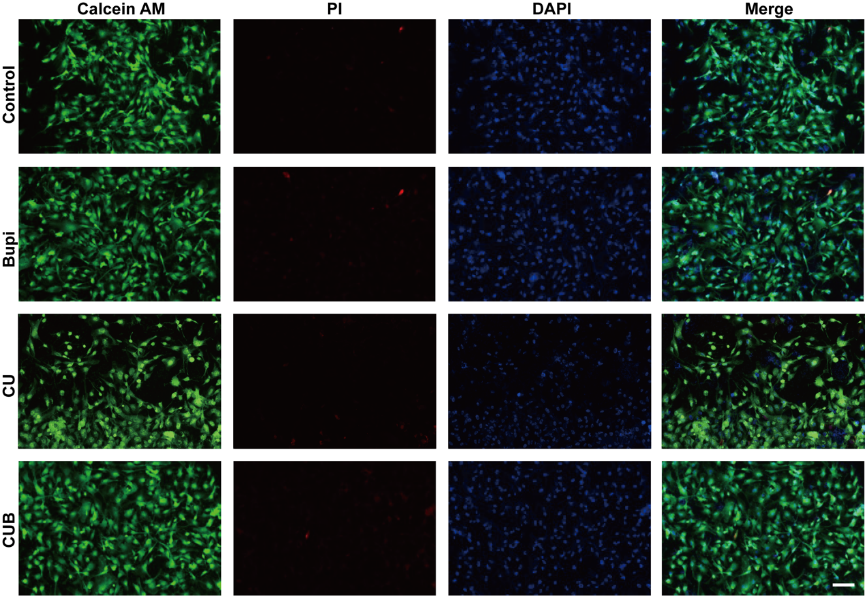
Figure S7.** Live/dead staining of astrocyte after co-cultured for 72 h. Scale bar = 100 μm.


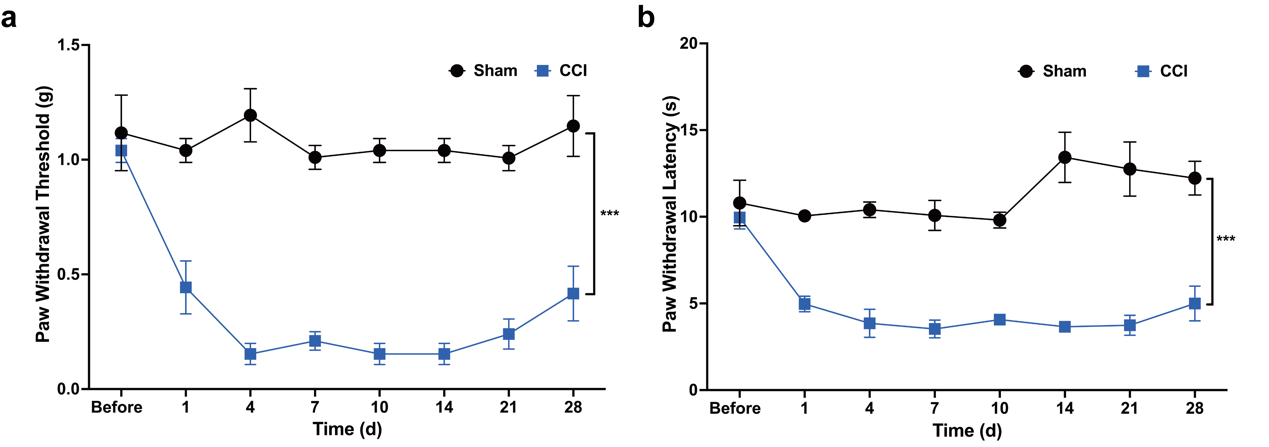


**Figure S8.** a) Baseline of mechanical paw withdrawal threshold before and after CCI model. b) Baseline of thermal paw withdrawal latency before and after CCI model.


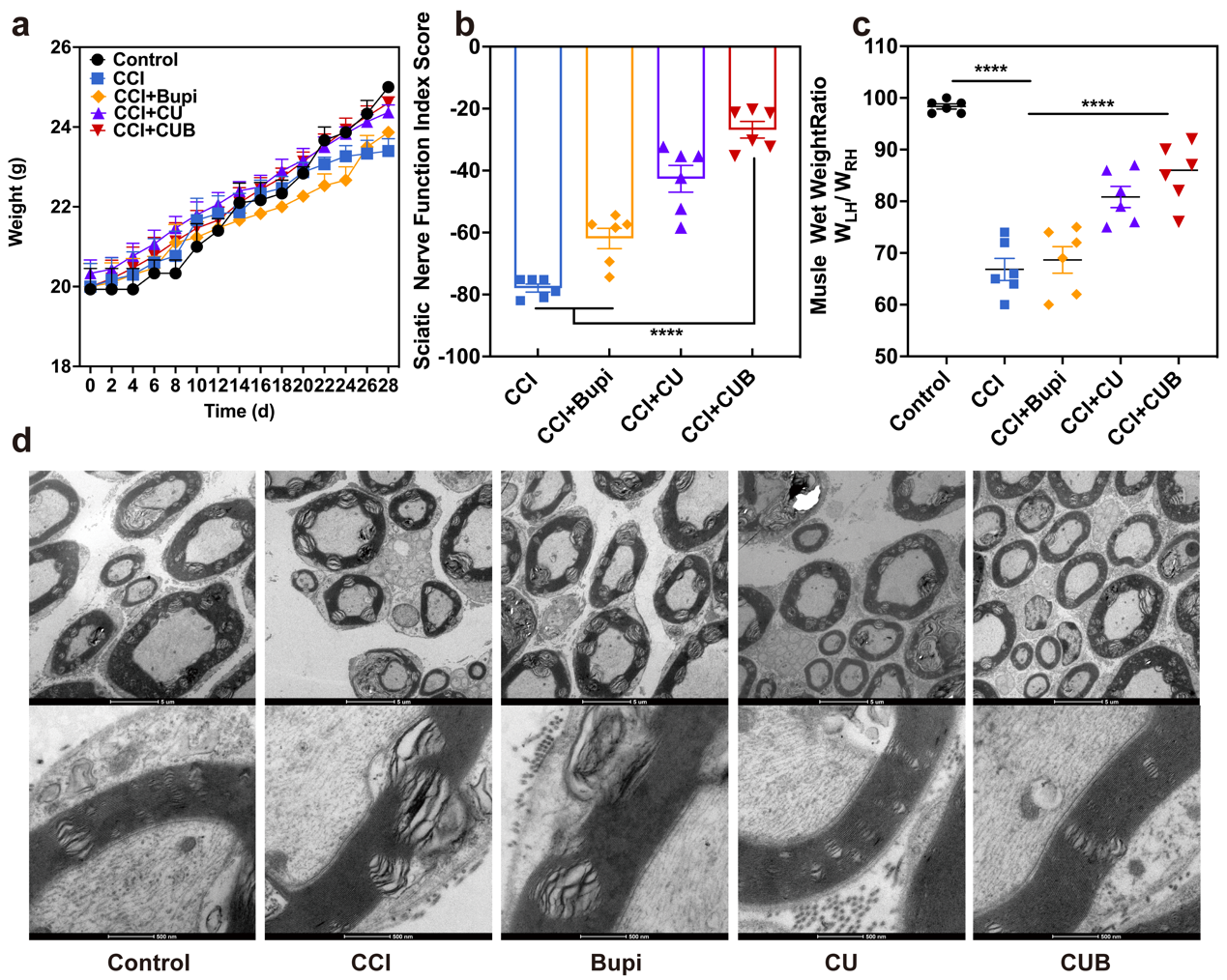


**Figure S9.** a) Weight of different groups mice in 28 days. Five groups: Control, CCI, CCI+Bupi, CCI +CU and CCI+CUB. b) SFI values of the sciatic nerves were calculated from a walking footprint. c) Muscle wet weight ratio of different treatment groups. d) TEM images of transverse section of sciatic nerve. All data are expressed as the mean ± s.e.m. **P*< 0.05, ***P* < 0.01, ****P*< 0.001, *****P* < 0.0001.

Control CCI Bupi


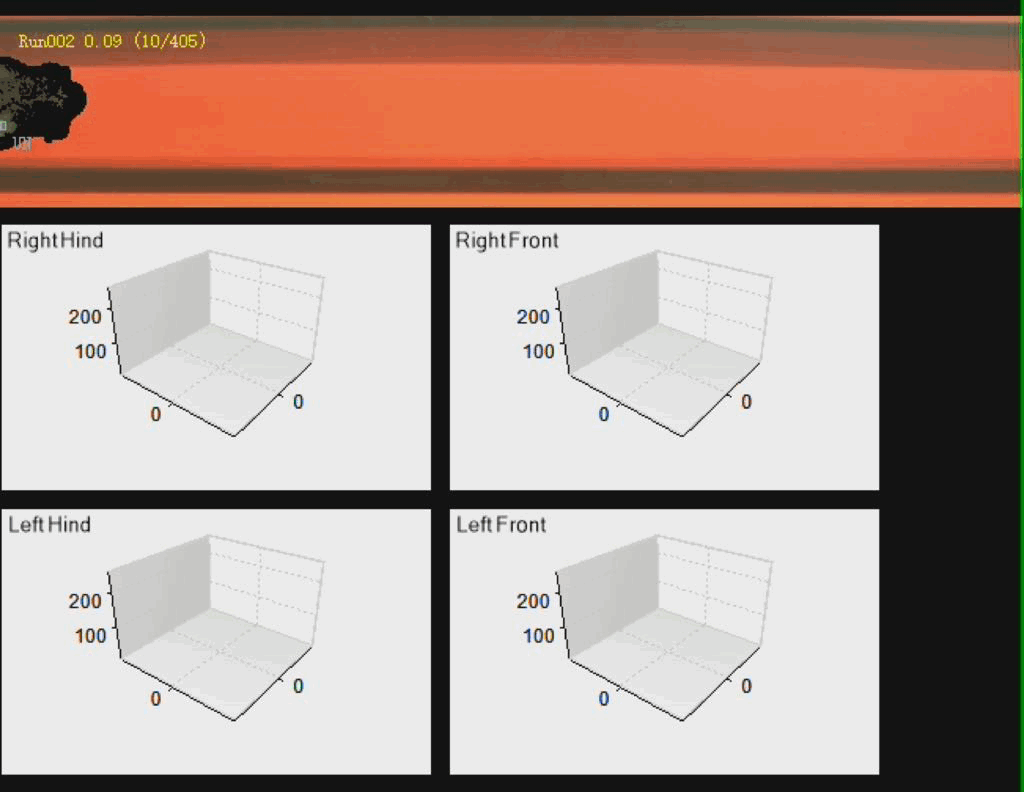

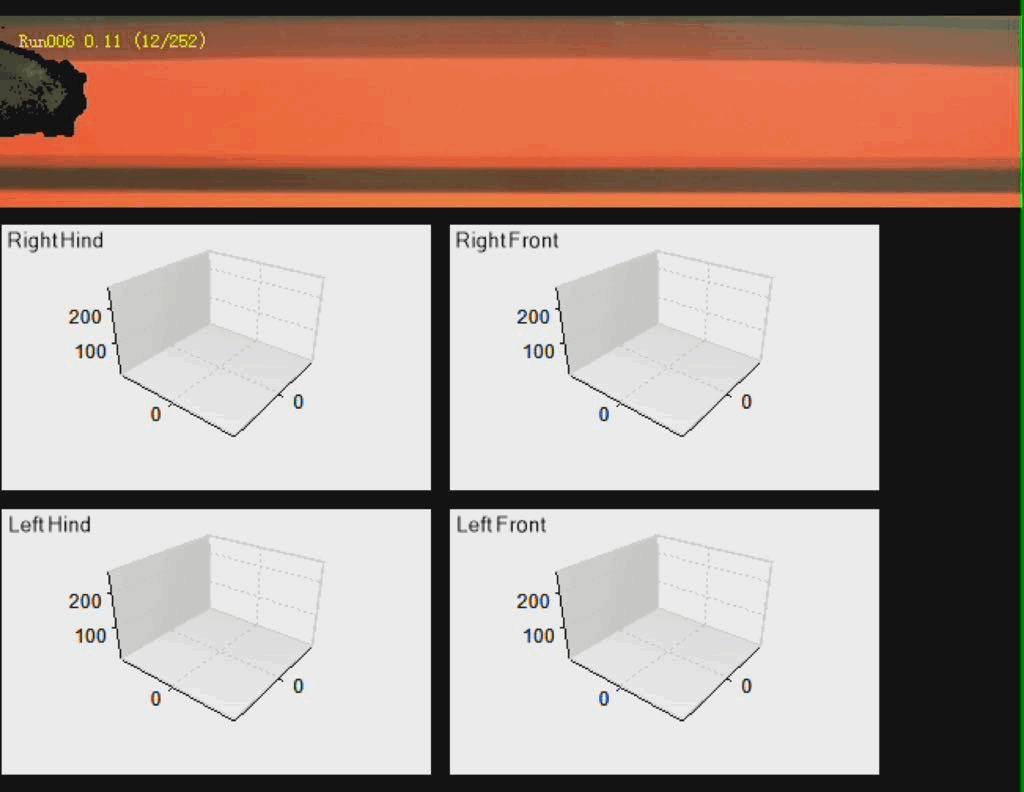

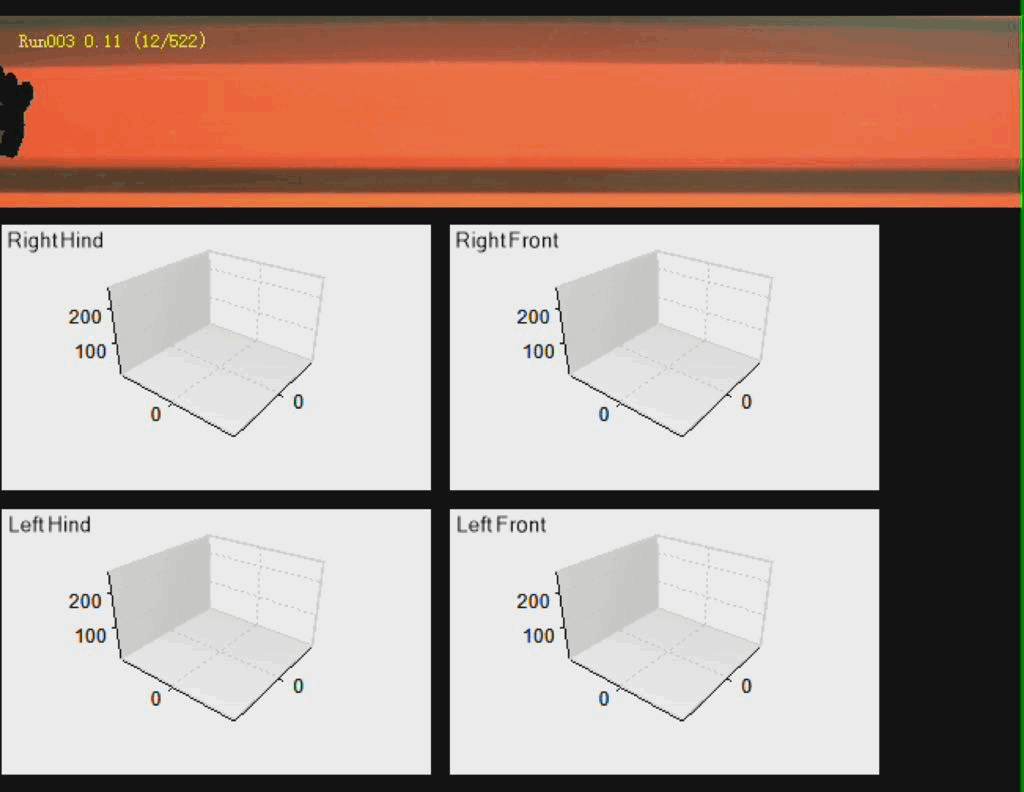


CU CUB


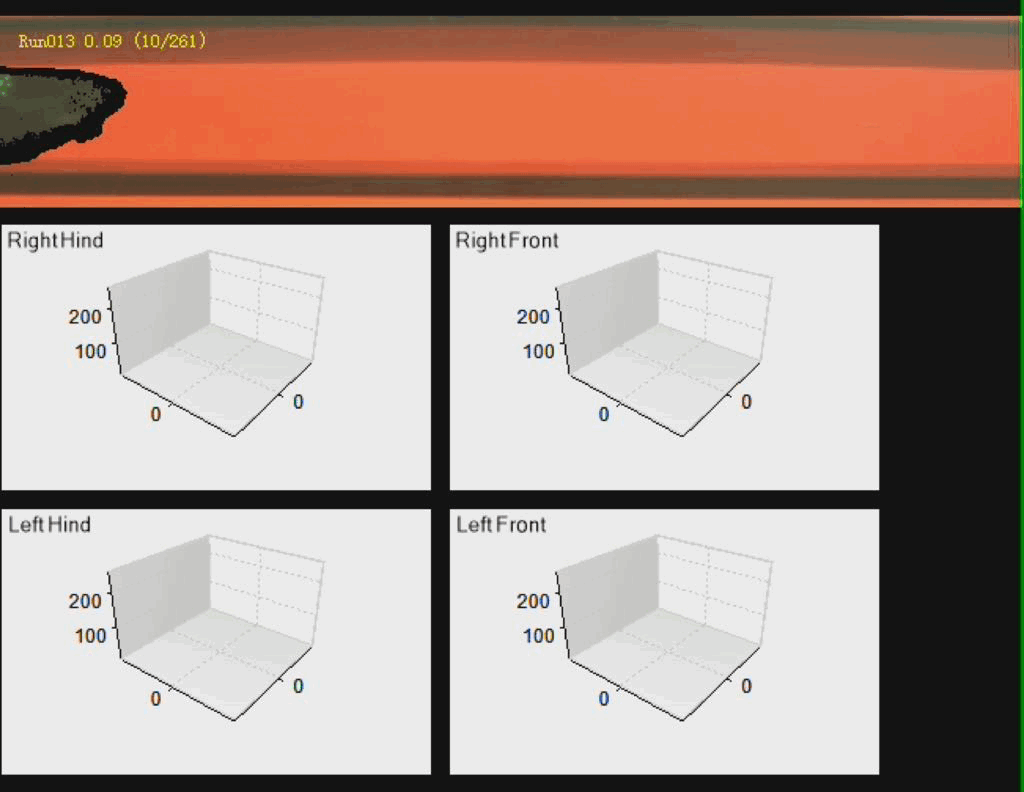

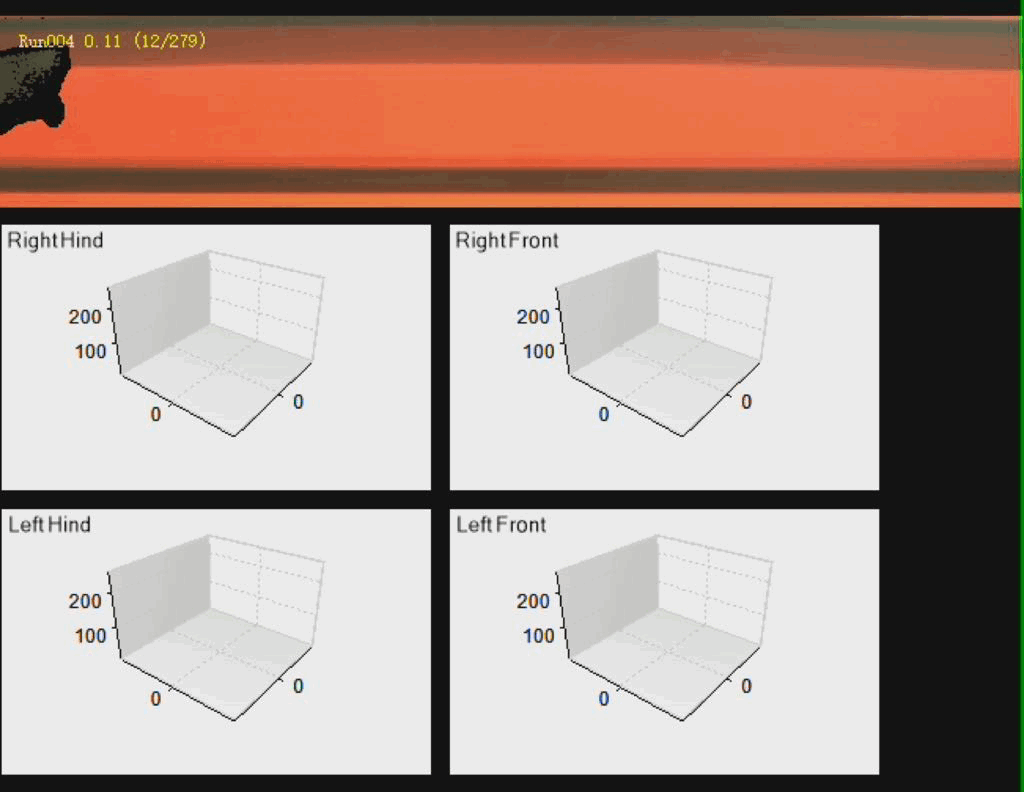


**Figure S10.** The pressure–time image and of Control, CCI, Bupi, CU and CUB groups.


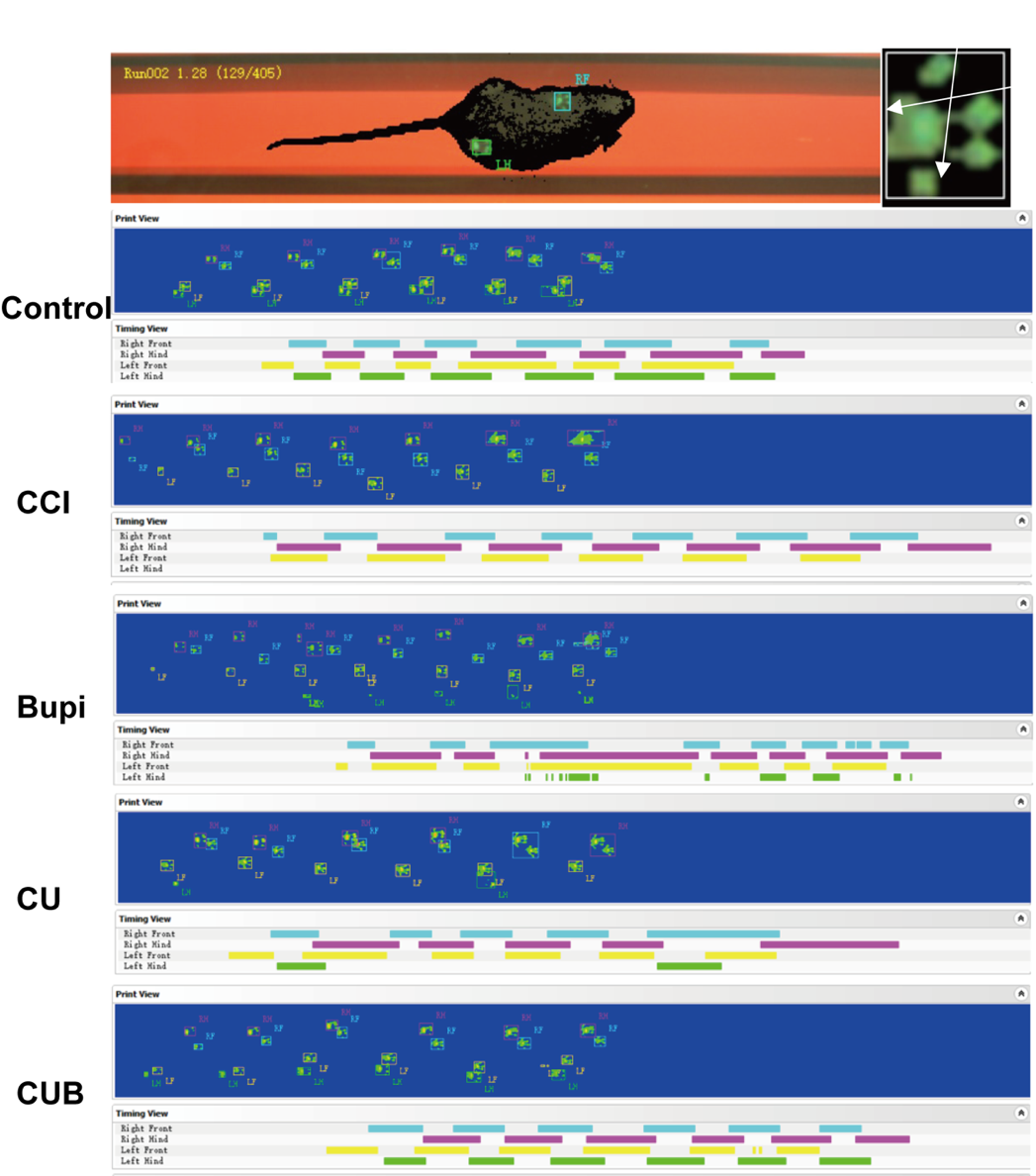


**Figure S11.** The thermal image of Control, CCI, Bupi, CU and CUB groups.


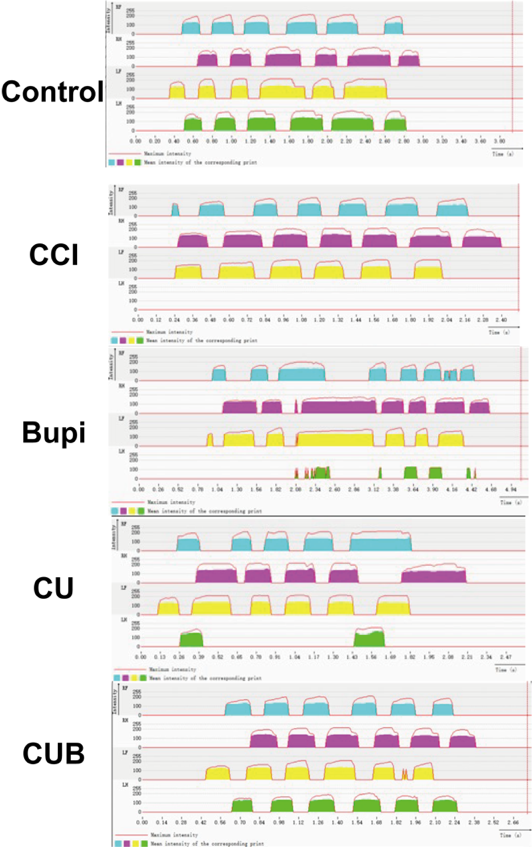


**Figure S12.** The gait pattern of Control, CCI, Bupi, CU and CUB groups.


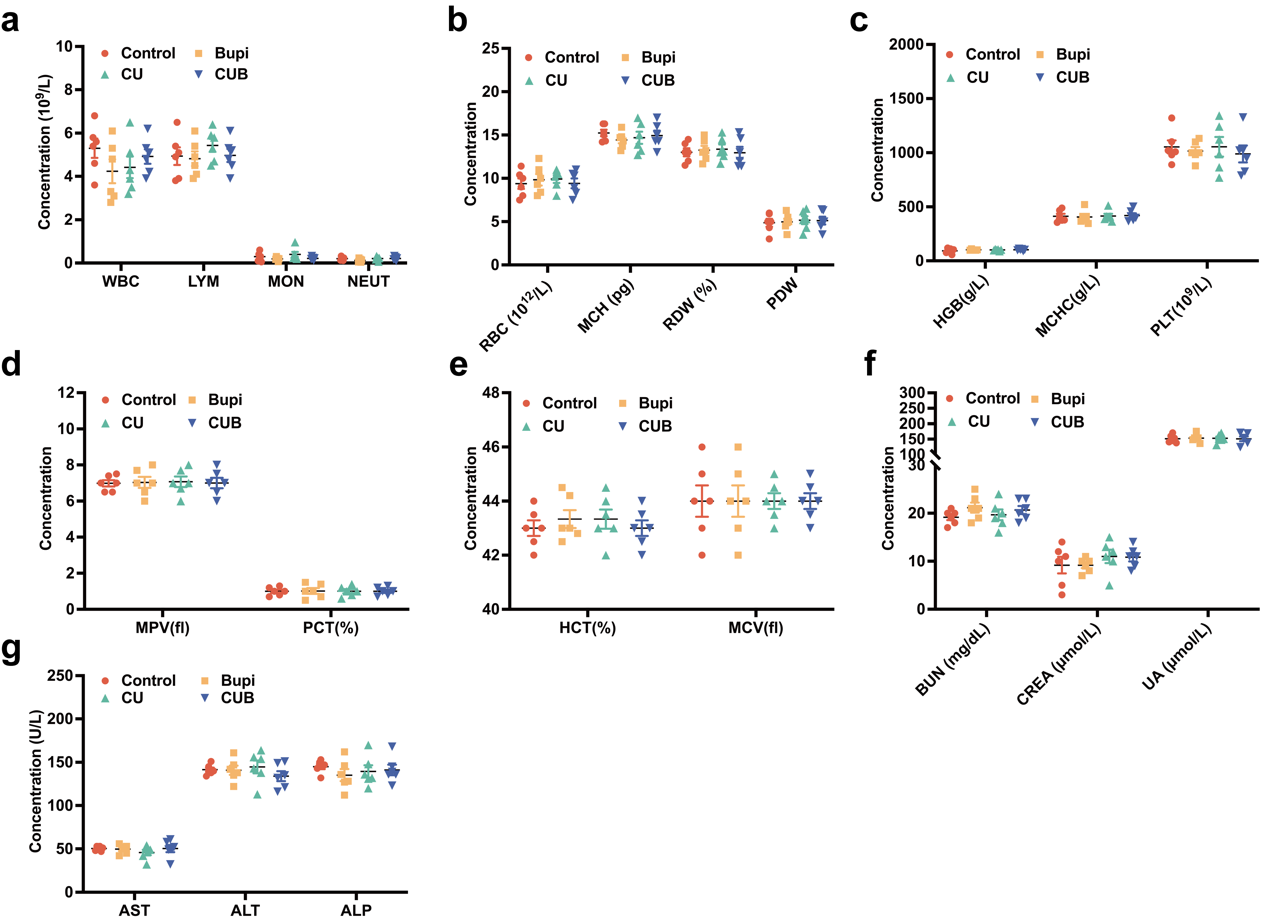


**Figure S13.** Safety and toxicity assessment of Bupi, CU, CUB after administration at 28^th^ day. a-e) Blood parameters of mice in control, Bupi, Cu and CUB groups. f) Serum levels of BUN, CREA, and UA of mice in control, Bupi, Cu and CUB groups. g) Serum levels of ALT, AST, and ALP of mice in control, Bupi, Cu and CUB groups.


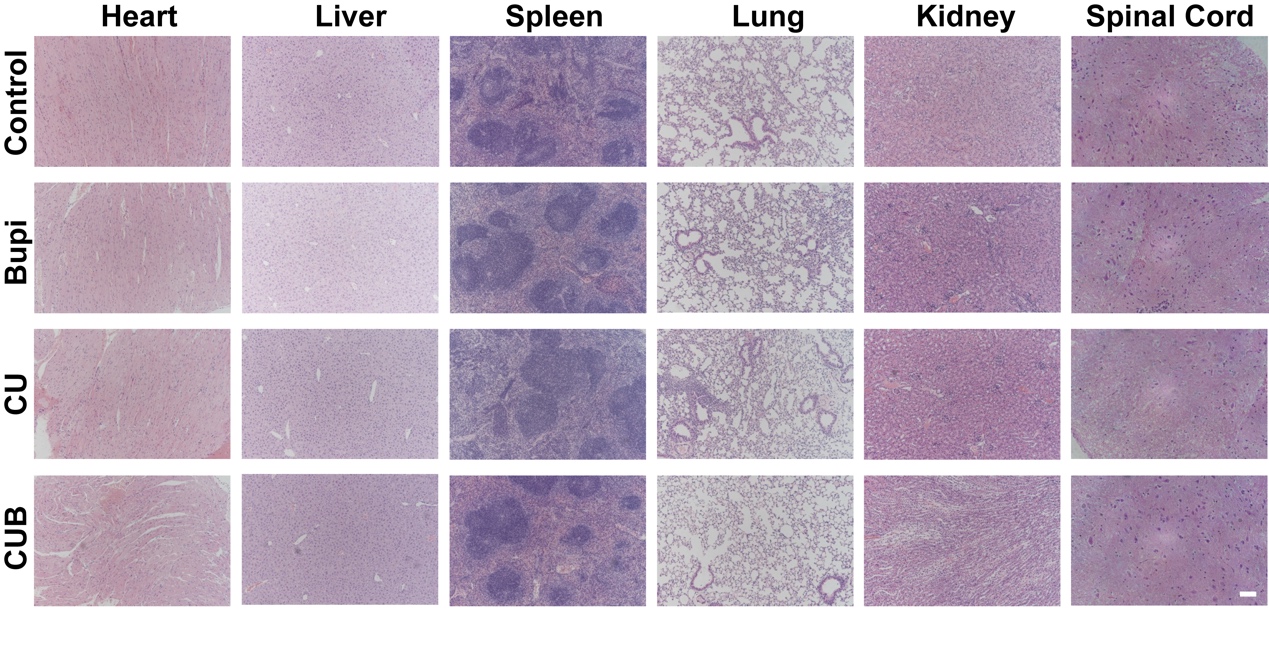


**Figure S14.** H&E staining sections of major organs to assess *in vivo* safety after injection of Bupi, CU, CUB at 28^th^ days. Scale bar = 100 μm.


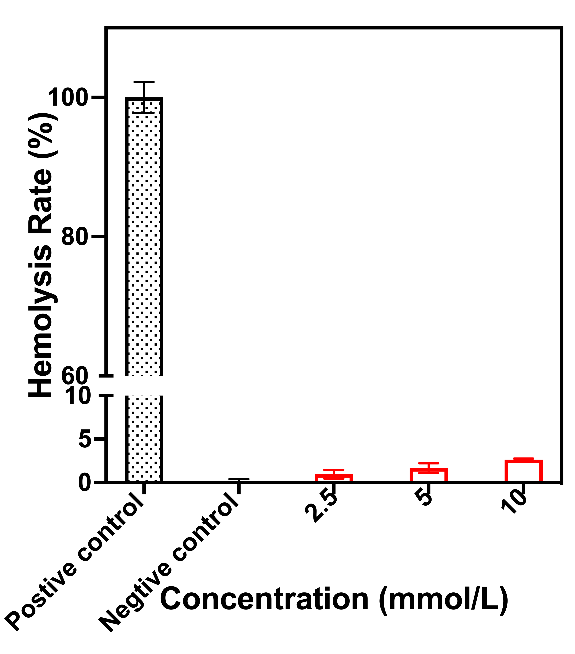


**Figure S15.** Hemolytic test with different concentrations of CUB.


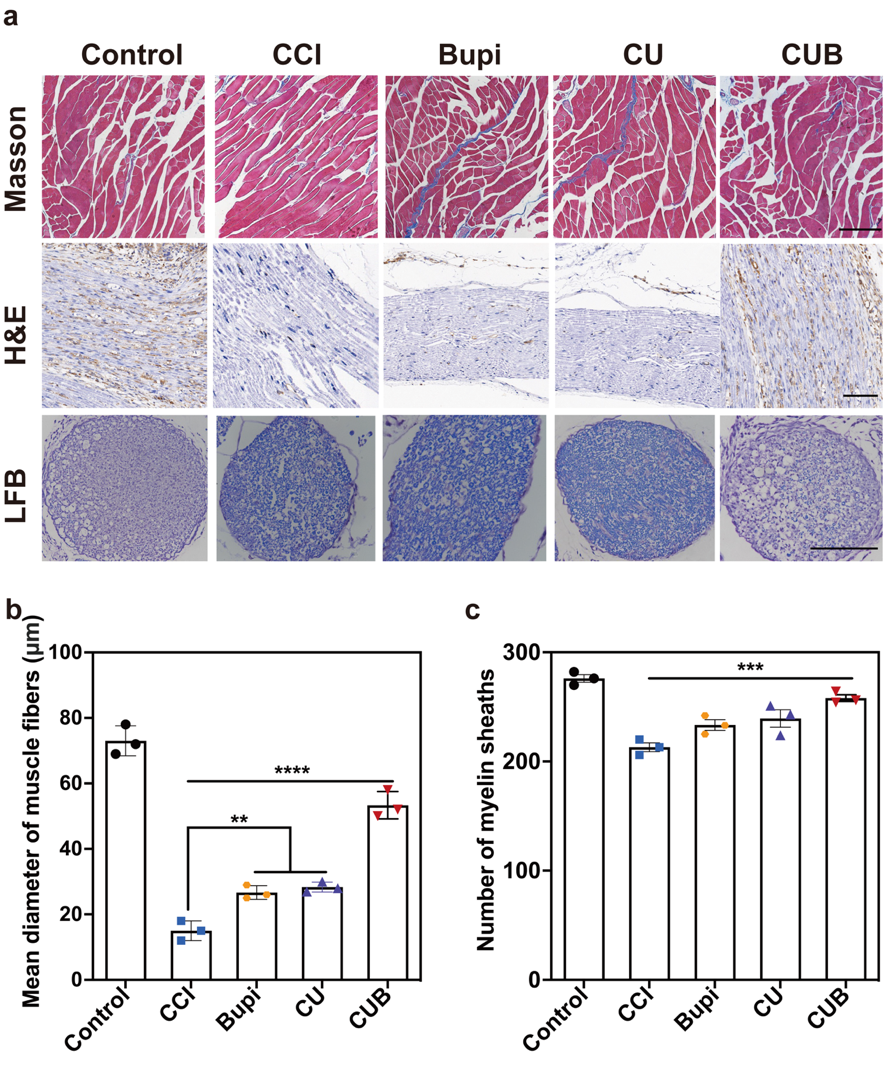


**Figure S16.** a) Masson staining of gastrocnemius muscle, red: muscle fibers; blue: collagen fibers, H&E staining of longitudinal section of sciatic nerve and Luxol Fast Blue (LFB) staining of transverse section of sciatic nerve. b) Statistical analysis of mean diameter of gastrocnemius muscle fibers. c) Number of myelin sheaths of transverse section of sciatic nerve. **P*< 0.05, ***P* < 0.01, ****P*< 0.001, *****P* < 0.0001. All scale bars = 100 μm.


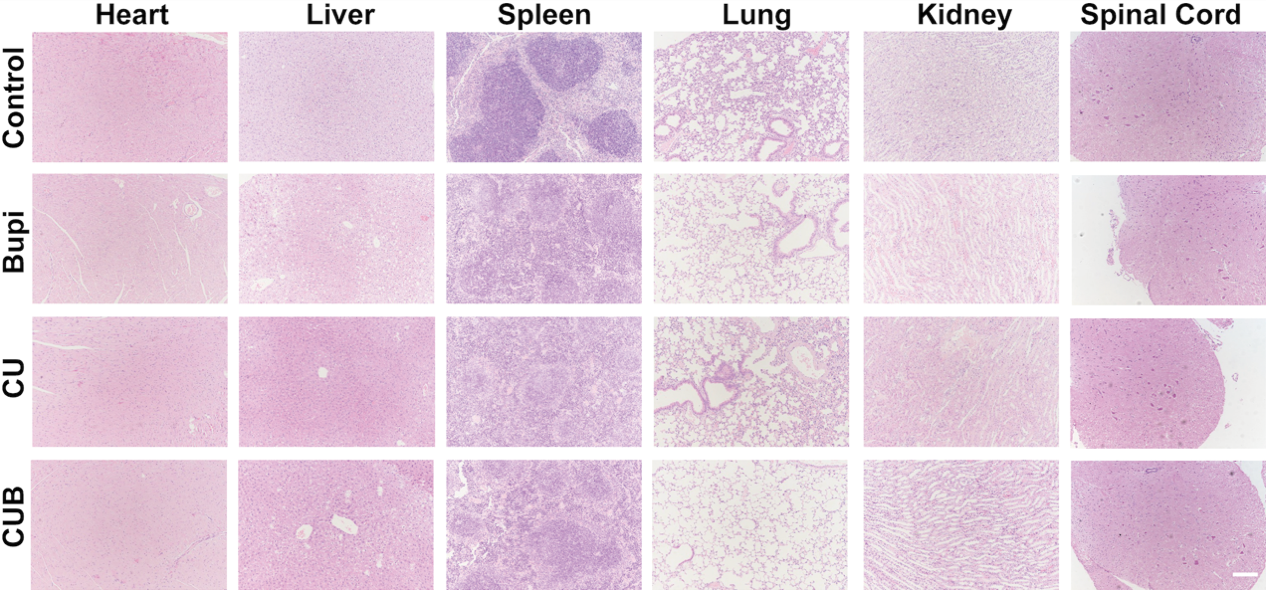


**Figure S17.** H&E staining sections of major organs to assess in vivo safety after injection of Bupi, CU, CUB after 12 weeks. Scale bar = 100 μm.


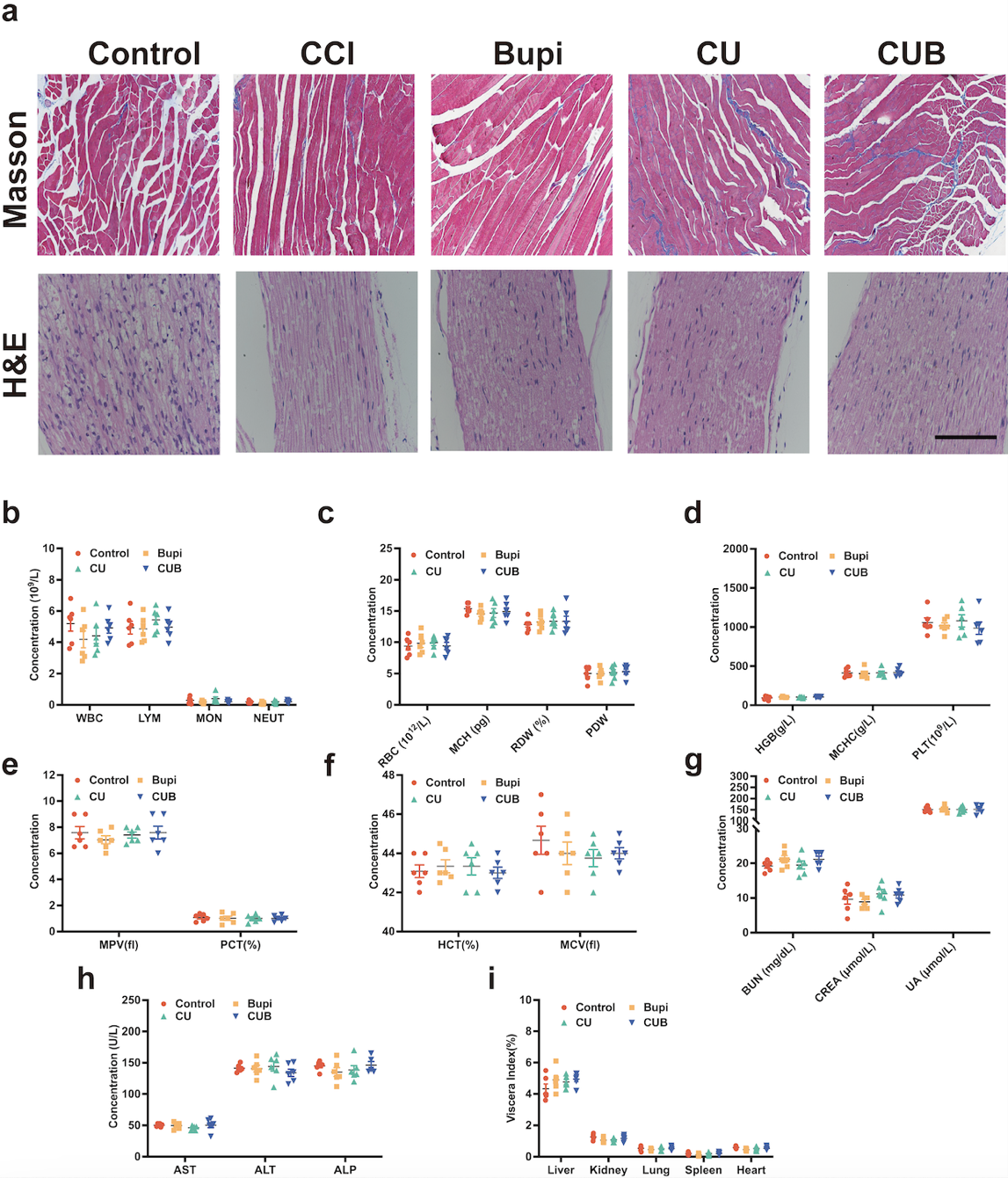


**Figure S18**. a) Masson staining of gastrocnemius muscle after 12 weeks, red: muscle fibers; blue: collagen fibers, H&E staining of longitudinal section of sciatic nerve. b-f) Blood parameters of mice in control, Bupi, Cu and CUB groups. g) Serum levels of BUN, CREA, and UA of mice in control, Bupi, Cu and CUB groups. h) Serum levels of ALT, AST, and ALP of mice in control, Bupi, Cu and CUB groups. i) Viscera index of mice in control, Bupi, Cu and CUB groups.


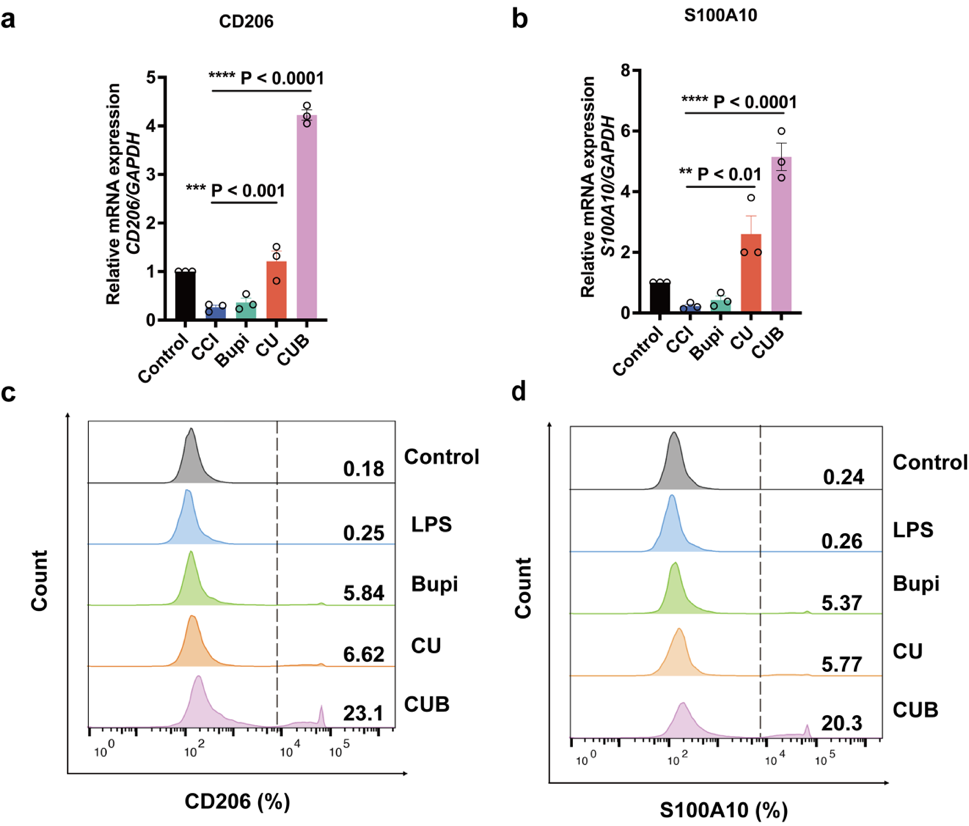


**Figure S19.** a-b) Relative mRNA expression levels of CD206 and S100A10, while c-d) present flow cytometric histograms for these same markers. These markers were assessed in different treatment groups, including Control, LPS (as a pro-inflammatory stimulus), Bupi, CU, and CUB. **P* < 0.05, ***P* < 0.01, ****P* < 0.001, *****P*< 0.0001.


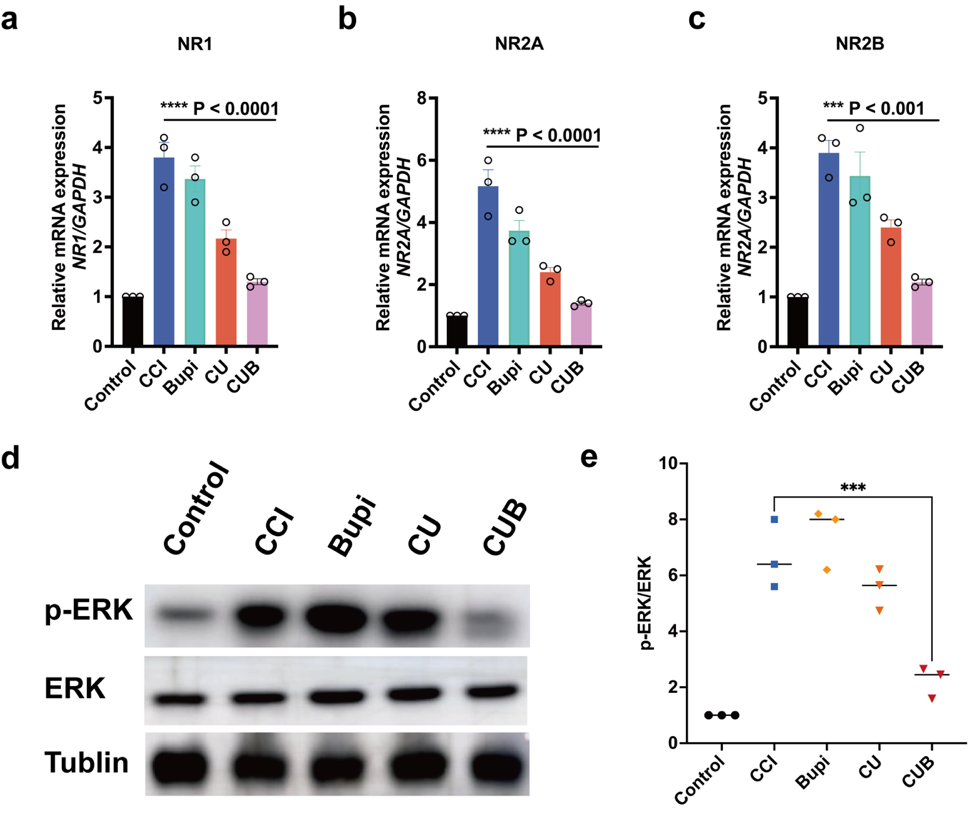


**Figure S20.** a-c) Relative mRNA expression levels of the major functional subunits of the NMDA receptor (NR1, NR2A, and NR2B) in the spinal dorsal horn across five experimental groups: Control, CCI, Bupi, CU, and CUB. d). Representative western blot data for p-ERK and total ERK, with tubulin as the loading control, while e) quantifies p-ERK/ERK ratios. **P* < 0.05, ***P* < 0.01, ****P* < 0.001, *****P*< 0.0001.

**Supplementary Table**

Supplementary Table1. Primers used in this study.

| Mouse GAPDH For | CAGTGGCAAAGTGGAGATTGTTG |
| --- | --- |
| Mouse GAPDH Rev | TCGCTCCTGGAAGATGGTGAT |
| Mouse IL-6 For | CTGCAAGAGACTTCCATCCAG |
| Mouse IL-6 Rev | AGTGGTATAGACAGGTCTGTTGG |
| Mouse IL-1β For | GCAACTGTTCCTGAACTCAACT |
| Mouse IL-1β Rev | AGTGCTGCCTTGCTGTTCTTGAG |
| Mouse TNF-α For | CAGGCGGTGCCTATGTCTC |
| Mouse TNF-α Rev | CGATCACCCCGAAGTTCAGTAG |
| Mouse IL-10 For | GCTGGACAACATACTGCTAACC |
| Mouse IL-10 Rev | ATTTCCGATAAGGCTTGGCAA |
| Mouse Arg-1 For | CTCCAAGCCAAAGTCCTTAGAG |
| Mouse Arg-1 Rev | AGGAGCTGTCATTAGGGACATC |
| Mouse C3 For | GAGCGAAGAGACCATCGTACT |
| Mouse C3 Rev | TCTTTAGGAAGTCTTGCACAGTG |
| Mouse PTX3 For | CGCAGGTTGTGAAACAGCAAT |
| Mouse PTX3 Rev | GGGTTCCACTTTGTGCCATAAG |
| Mouse CCL2 For | TTAAAAACCTGGATCGGAACCAA |
| Mouse CCL2 Rev | GCATTAGCTTCAGATTTACGGGT |
| Mouse CD206 For | CTCTGTTCAGCTATTGGACGC |
| Mouse CD206 Rev | TGGCACTCCCAAACATAATTTGA |
| Mouse S100A10 For | GCTTACGTTTCACAGGTTTGC |
| Mouse S100A10 Rev | AAGCCCACTTTGCCATCTCG |
| Mouse NR1 For | TGCCCTCCAAGTTCATCTTTC |
| Mouse NR1 Rev | GGCTGAGCTGGCTATGTTGAG |
| Mouse NR2A For | ACGTGACAGAACGCGAACTT |
| Mouse NR2A Rev | TCAGTGCGGTTCATCAATAACG |
| Mouse NR2B For | CAGCAAAGCTCGTTCCCAAAA |
| Mouse NR2B Rev | GTCAGTCTCGTTCATGGCTAC |
